# Tuning of cortical color mechanisms revealed using steady-state visually evoked potentials

**DOI:** 10.1162/IMAG.a.130

**Published:** 2025-08-28

**Authors:** Ana Rozman, Dylan J. Watts, Lucy P. Somers, Bora Gunel, Chris Racey, Katie Barnes, Jenny M. Bosten

**Affiliations:** School of Psychology, University of Sussex, Brighton, United Kingdom

**Keywords:** color vision, SSVEP, EEG, tuning, cortical color mechanisms

## Abstract

Color information is thought to enter the cortex via two dominant retinogeniculate pathways, one signaling teal to red, and the other violet to lime color variation. The cortex is thought to transform this representation, but the properties of human cortical color mechanisms are not very well understood. In four experiments, we characterized the tuning of cortical color mechanisms by measuring the intermodulation of steady-state visually evoked potentials (SSVEPs), thought to index the extent to which shared neural resources process stimuli flickering at different frequencies. Stimuli were isoluminant chromatic checkerboards where odd and even checks flickered at different frequencies. As hue dissimilarity between odd and even checks increased, the amplitude of an intermodulation component (I_1_) at the sum of the two stimulus frequencies decreased, revealing color tuning functions. In Experiment 1, we found similar broad tuning functions for “cardinal” and intermediate color axes, implying the action of intermediately tuned cortical color mechanisms. In Experiment 2 we found similar tuning functions for “checkerboards” without perceptible edges because the checks were formed from single pixels (~0.096°), implying that the underlying neural populations do not rely on spatial chromatic edges. In Experiment 3 we found consistent color tuning functions across check sizes. In Experiment 4 we measured full 360° tuning functions and found results compatible with opponent color responses. The observed cortical color tuning functions are consistent with those measured using psychophysics and electrophysiology, implying that the method is useful for investigating color representations in the brain.

## 1 Introduction

Research on the neural mechanisms of human color vision has led to a relatively good understanding of color representation in retinogeniculate pathways. However, cortical color mechanisms are less well understood. Though the tuning of cortical color mechanisms has been studied using electrophysiology in animals, and using psychophysics in humans, there have been few direct measurements of human cortical color tuning functions acquired by neuroimaging. We used a new approach, measuring steady-state visually evoked potentials (SSVEPs) to characterize the tuning functions of cortical color mechanisms in humans.

Normal human color vision begins with the relative activations of three types of retinal cone photoreceptors, sensitive to short (S), medium (M), or long (L) wavelengths of light. Cone signals are then thought to be combined postreceptorally by distinct populations of retinal bipolar cells, setting up “cardinal” color pathways which remain segregated in the retina and LGN, and at least as far is the input layers of V1. The S/(L+M) pathway carried by small bistratified ganglion cells and through koniocellular layers of the LGN signals color differences between lime green and violet. The L/(L+M) pathway carried by the midget retinal ganglion cells and through parvocellular layers of the LGN signals color differences between teal and red (Conway et al., 2018; Solomon & Lennie, 2007). There is debate over how far color information carried by the different “cardinal” retinogeniculate pathways remains segregated in the cortex (Gegenfurtner, 2003; Goddard et al., 2010; Nunez et al., 2021), and over the number and tuning bandwidths of cortical color mechanisms (Emery et al., 2017; Gegenfurtner, 2003). Another unresolved question about cortical color representation is to what extent it is bipolar (where two “opponent” hues are represented with the achromatic point represented somewhere in the middle of the mechanism’s response range), or unipolar (where color variation between a single hue and the achromatic point is represented by each mechanism). Chromatic signals in the input layers of V1 must inherit the bipolar color representations of the retinogeniculate color pathways, and there is evidence for bipolar color tuning in macaque single neurons even beyond V1 (Kiper et al., 1997). However, psychophysical evidence (Emery et al., 2017, 2023; Malkoc et al., 2005) and other electrophysiological evidence (Gegenfurtner, 2003; Solomon & Lennie, 2007) is consistent with multiple “monopolar” color mechanisms, each tuned to a particular hue and responding to neighboring hues, within a given bandwidth.

Human psychophysical studies of cortical color mechanisms have typically found broad tuning (Lindsey & Brown, 2004; Sankeralli & Mullen, 1997), especially when “off-axis looking” is minimized (D’Zmura & Knoblauch, 1998). Primate electrophysiology has found diverse hue preferences in color-selective neurons in V1 (e.g., Hass & Horwitz, 2013), V2 (e.g., Solomon & Lennie, 2005), V3 (Gegenfurtner et al., 1997), V4 (Bohon et al., 2016), and downstream color-selective areas (Conway et al., 2007; Komatsu et al., 1992), and has revealed tuning functions with a range of half-amplitude bandwidths from 30° to 170° in hue angle in V1 (Wachtler et al., 2003) and from 20° to 85° in V2 (Kiper et al., 1997).

Many cortical color-selective neurons are jointly tuned to color and other stimulus properties, for example, to luminance (Johnson et al., 2001; Schluppeck & Engel, 2002), spatial frequency (Lennie et al., 1990), and orientation (Johnson et al., 2008; Leventhal et al., 1995). A distinction has been drawn between so-called “double-opponent” neurons which respond strongly to chromatic edges but weakly to full-field chromatic stimuli, and “single-opponent” neurons that show the opposite pattern (Conway, 2001; De & Horwitz, 2021; Johnson et al., 2008; Livingstone & Hubel, 1984; Shapley & Hawken, 2011). Whether or not these two types of cell are constituents of different chromatic pathways is not known: Single-opponent cells may be intermediates in wiring double-opponent cells (Conway & Livingstone, 2006). The variation in spatial preferences among color-responsive neurons implies that color tuning may vary with the spatial properties of stimuli if they target different neural populations.

One existing study by Chen and Gegenfurtner (2021) used neuroimaging to characterize human cortical color tuning, by measuring SSVEPs. SSVEPs occur in response to flickering visual stimuli (Norcia et al., 2015; Regan, 1966), and are typically analyzed in the frequency domain where EEG noise is segregated into many frequency bands, providing high signal-to-noise ratios (SNRs) for brain responses at the stimulus frequency (Rossion & Boremanse, 2011). Chen and Gegenfurtner (2021) based their method on chromatic noise masking (Gegenfurtner & Kiper, 1992), where chromatic contrast in a target was masked by surrounding chromatic noise, to a degree dependent on the color difference between target and surround chromaticities. They found broad color tuning functions for both cardinal and intermediate color axes (see Peterzell & Norcia, 1997 for analogous work investigating spatial frequency tuning).

We investigate the potential of intermodulation of SSVEPs as a way of measuring cortical tuning functions (Regan & Regan, 1987). Intermodulation components can arise when concurrently presented stimuli flicker at different, independent frequencies (e.g., F_1_ and F_2_), and occur in the frequency domain at the sum or difference of combinations of the stimulus frequencies and their harmonics (e.g., 1F_1_+1F_2_, 2F_2_-1F_1_). Because there are no modulations of the stimulus at intermodulation frequencies, intermodulation components do not reflect direct responses to stimulus variation at these frequencies. Rather, they are thought to arise in the brain due to nonlinear interactions between responses to the two stimulus frequencies in the same neural populations. They are, therefore, thought to index the degree to which shared neural resources process both of the stimuli (Gordon et al., 2019; Regan & Regan, 1989). Our group has previously compared the efficiency of using intermodulation components with the efficiency of using a reverse oddball-based SSVEP method for measuring cortical color tuning functions (Rozman et al., 2025).

We presented flickering checkerboards where odd checks flickered at one frequency (F_B_) between gray and a fixed hue across trials, and even checks flickered at a different frequency (F_A_) between gray and a hue that varied between trials. We expected that amplitudes of intermodulation components would be maximal when odd and even checks have the same chromaticity (and are, therefore, processed by the same color mechanisms), and would decrease with the increasing color difference between odd and even checks, allowing us to plot cortical color tuning functions. We measured tuning functions based on the I_1_ intermodulation component (at 1F_A_+1F_B_) for cardinal and intermediate color axes (Experiment 1), for stimuli lacking perceptible chromatic edges (Experiment 2), as a function of the size of checkerboard checks (Experiment 3), and around the full hue circle (Experiment 4). Our results reveal (i) similar tuning bandwidths for cardinal and intermediate axes, implying the action of intermediate color mechanisms; (ii) that, surprisingly, tuning functions do not depend on perceptible chromatic edges, suggesting that the underlying color mechanisms are not spatially selective; and (iii) that there is a second peak in tuning for opponent colors, compatible with a bipolar color representation.

To allow a comparison between our results and those of Chen and Gegenfurtner’s (2021) study, we also plot tuning functions based on SSVEPs at the frequency of the checks with fixed chromaticity across conditions (1F_B_). SSVEP amplitudes at this frequency are thought to be modulated by chromatic masking, depending on the hue difference between the fixed and variable hues. When the fixed and variable hues are similar, chromatic masking is strong and amplitudes at 1F_B_ (the frequency at which the odd checks flicker between gray and a fixed hue) are expected to be low. When the fixed and variable hues are very different, chromatic masking is weak and, therefore, amplitudes at 1F_B_ are expected to be high. We thus expect opposite relationships between SSVEP amplitudes and hue differences between the fixed and variable hues for I_1_ versus 1F_B_.

## 2 General Methods

Provided in this section are methods common to all four experiments.

### 2.1 Participants

Participants in all experiments had normal color vision assessed using an “Anomaloskop” anomaloscope (Oculus, Wetzlar, Germany; Experiments 1–3) or the Ishihara Plates Test (Experiment 4), and were recruited via email or word-of-mouth. Participants provided written informed consent and were compensated £10 per hour for their time. The study received ethical approval from the University of Sussex Science and Technology Cross Schools Research Ethics Committee (ER/DJW41/1), and adhered to the tenets of the World Medical Association’s Declaration of Helsinki (2013) with the exception that it was not pre-registered.

### 2.2 Equipment

Electroencephalographic data were acquired using 64-channel Waveguard caps (ANT Neuro, Hengelo, Netherlands) via a high-speed 64-channel ANT Neuro amplifier sampling at 1000 Hz. EEG data were recorded in asa-lab™ (ANT Neuro, Hengelo, Netherlands). Additional electrodes were used to measure horizontal (HEOG) and vertical (VEOG) eye movements. Since the SSVEP method relies on extremely precise timing at the level of individual display frames, a custom photodiode input to the EEG amplifier was fitted to check stimulus timing.

Stimuli were displayed on a GDM FW900 CRT monitor (Sony, Tokyo, Japan). The monitor was gamma corrected using a luminance meter (Konica-Minolta, Tokyo, Japan) and color calibrated using a PR650 SpectraScan spectroradiometer (PhotoResearch, Chatsworth, CA, USA). Stimuli were presented using a ViSaGe MKII Stimulus Generator (Cambridge Research Systems, Rochester, UK) using Matlab 2017 (The MathWorks, Natick, MA, USA). Triggers were sent to the EEG amplifier from the ViSaGe via BNC and parallel port connections. The monitor had a resolution of 800 x 600 pixels and ran at a frame rate of 160 Hz.

### 2.3 Heterochromatic flicker photometry

Heterochromatic flicker photometric measurements were made to calculate stimuli that were isoluminant for individual participants. Stimuli consisted of a 2D annulus with an outer diameter of 41.9° and inner diameter of 5.7°, which oscillated at 11.4 Hz between two colors. On each trial, the intensity of one color was fixed, and participants adjusted the intensity of the other color until they perceived minimum flicker (the point of isoluminance). On the basis of these measurements, stimuli in the four experiments were made isoluminant to ensure that they isolated chromatic mechanisms and did not stimulate luminance mechanisms.

### 2.4 Stimuli

**Fig. 1.**
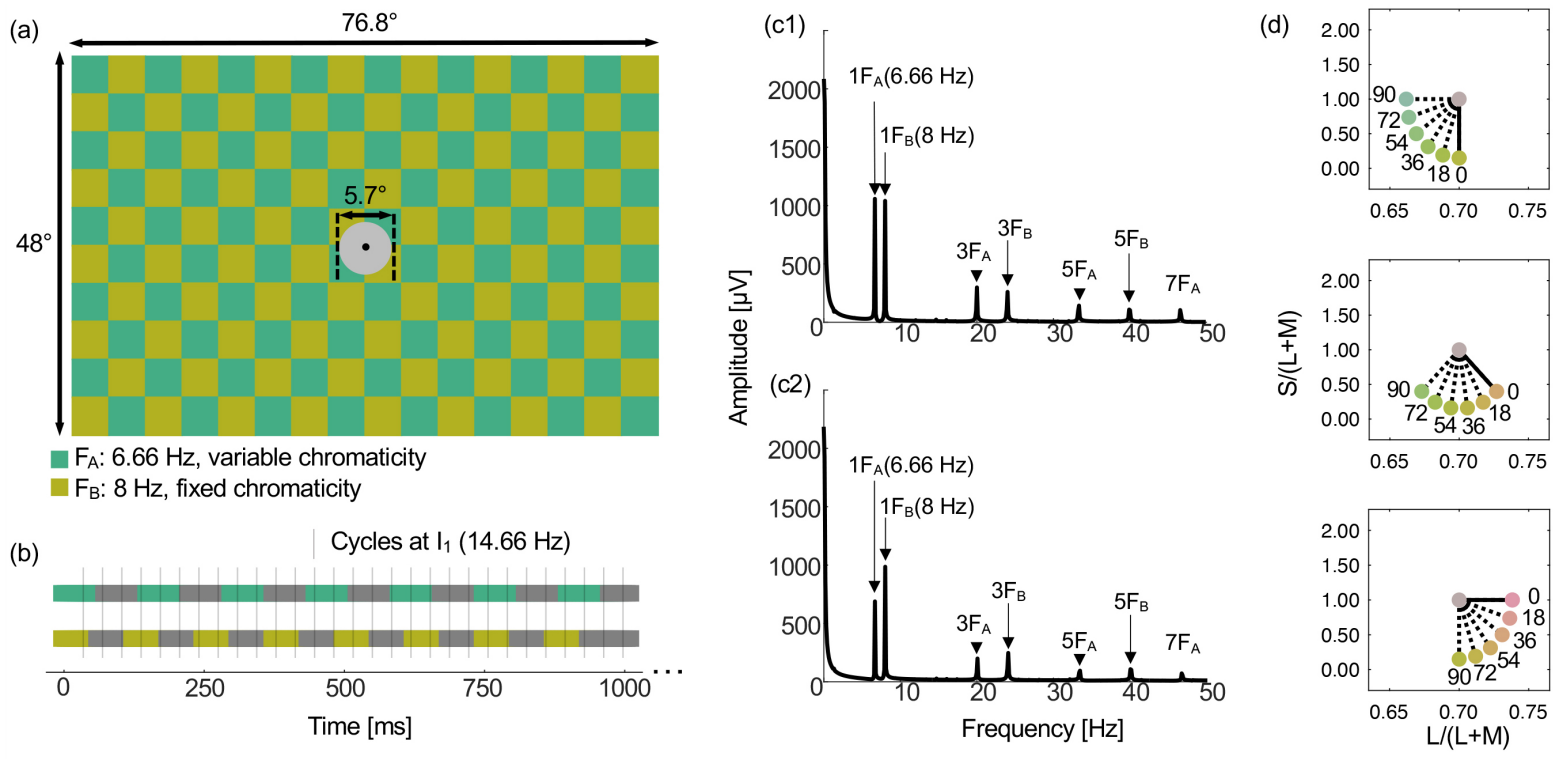
Stimulus properties. (a) Stimuli were isoluminant chromatic flickering checkerboards. Even checks flickered at F_A_ (6.66 Hz) between a gray metameric with equal energy white and a “variable” chromaticity that depended on the color condition. Odd checks flickered at F_B_ (8 Hz) between a gray metameric with equal energy white and a “fixed” chromaticity. A central 5.7° disk was metameric with equal energy white and did not flicker. (b) Representation of the stimulus modulation over time. Odd checks modulated through a square wave between equal energy white and the fixed chromaticity at 8 Hz. Even checks modulated through a square wave between equal energy white and a variable chromaticity at 6.66 Hz. Marked on the figure by thin vertical gray lines are cycles at I_1_ (14.66 Hz), showing that there is no consistent modulation of the stimulus occurring at I_1_. (c1-c2) Photodiode amplitudes as a function of frequency for two randomly selected conditions in Experiment 4 (see Section 6). Each panel shows photodiode responses averaged over all 10 participants and all trials for the 30° hue angle difference condition (c1) and for the 180° hue angle difference condition (c2). Preprocessing (such as filtering) was as for the EEG data. Visible peaks up to 50 Hz are labeled within each plot. The figure confirms light modulation at the two stimulus modulation frequencies and their odd harmonics, but no light modulation in the stimulus at I_1_ or at other intermodulation frequencies. (d) Stimulus chromaticities were specified in a version of the MacLeod and Boynton (1979) chromaticity diagram based on the Stockman, MacLeod, and Johnson (1993) cone fundamentals. For Experiment 1, the fixed chromaticity was either a decrement along the S/(L+M) axis (lime green; upper panel), along an intermediate color axis (orange; middle panel), or an increment along the L/(L+M) axis (red; lower panel). In each panel, the modulation made between the fixed chromaticity and equal energy white (indicated by the gray disc at the center of each panel) is indicated by the solid line. For each fixed color axis, there were 6 color conditions, where the variable chromaticity differed from the fixed chromaticity in steps of 18° from 0° to 90°. Modulations between variable chromaticities and equal energy white are indicated by dashed lines. For Experiments 2 and 3, the S/(L+M) axis in a decremental direction was tested (upper panel). For Experiment 4, the fixed chromaticity was also a decrement along the S/(L+M) axis (upper panel), and there were 12 color conditions in 30° steps between 0° and 330° from the fixed chromaticity (not shown).

The stimuli for all four experiments consisted of flickering checkerboards extending 76.8° horizontally and 48° vertically across the entire CRT display (Fig. 1a), with a viewing distance of 30 cm. Odd checks flickered between a gray metameric with equal energy white and a fixed hue at 8 Hz, eliciting SSVEPs at 8 Hz and at 8 Hz harmonics. Even checks flickered between a gray metameric with equal energy white and a variable hue at 6.66 Hz, eliciting SSVEPs at 6.66 Hz and at 6.66 Hz harmonics. We chose not to counterbalance the fixed and variable hues across the two frequencies as we wanted to analyze results at 8 Hz for comparison with Chen and Gegenfurtner (2021), and thus did not want to confound effects of frequency and hue angle difference on SSVEP amplitudes. The chromatic flicker was through a square wave between a gray metameric with equal energy white and the edge of the maximum isoluminant saturation circle allowed by the monitor’s gamut (Fig. 1d). To avoid disruption to isoluminance by the macular pigment which selectively filters short wavelength light in the central part of the visual field (Hammond et al., 1997), we included a central steady (non-flickering) 5.7° disk metameric with equal energy white around a central black fixation point (Fig. 1a). The luminance of all stimuli was approximately 59 cd.m^-2^ but varied between participants depending on their individual heterochromatic flicker photometry settings.

### 2.5 Procedure

Participants first completed the heterochromatic flicker photometry task. The consistency of intensity settings was assessed, and if found to range >20%, the task was repeated. Participants were then fitted with facial electrodes and an EEG cap in accordance with the 10/20 system (Jurcak et al., 2007). We ensured that impedances for electrodes of interest were <10 kΩ, and for other electrodes <20 kΩ. In the experiment, participants viewed a series of 10.5-second trials (see details of methods for each separate experiment). Participants were instructed to maintain fixation on the central fixation dot during trials and to blink between trials, avoiding excessive blinking during trials. Participants controlled inter-trial intervals by pressing a button on a keyboard when they were ready for the next trial. Raw EEG data were monitored in real time for signs of fatigue or other issues. These could include excessive alpha band noise, general muscle noise, or muscle events from yawning. If such issues were identified, participants were encouraged to take a break. Participants were free to take rest breaks of any duration at any point in experimental sessions.

### 2.6 EEG Analysis: pre-processing

Pre-processing was conducted in MATLAB using Letswave 7 (https://letswave.cn/). First, raw EEG data were imported and mapped to the ANT neuro standard 64-channel electrode coordinate system. Raw data were visually inspected for excessive noise resulting from equipment failures. Problematic channels were removed. No exclusions of channels were made from our analysis cluster, consisting of Oz, O1, O2, Pz, P1, P2, P3, P4, POz, PO3, and PO4, chosen to allow us to best capture occipital activity. An overview of signals at other posterior and occipital channels is presented in Supplementary Information 1 (Supplementary Fig. S1). We found equivalent tuning functions for electrodes in different posterior/anterior bands, implying that they may all originate from the same source. This result is compatible with the low spatial resolution of EEG (Nunez & Westdorp, 1994), where sources of signals cannot be reliably inferred from electrode locations alone.

Signals were re-referenced to the grand average of all remaining cortical electrodes. In addition to our analysis cluster, channels FPz, HEOG, and VEOG were extracted to detect eye movements. A Butterworth filter for 50 Hz noise and a high-pass filter (>0.1 Hz included) were applied. Data were then segmented into trials using an epoch size of 9000 ms (from 1 to 10 seconds within the 10.5-second trial), which included an integer number of stimulus cycles at both flicker frequencies (Retter & Rossion, 2016; Retter et al., 2023). No ocular artifacts were removed from the data because we found that removing ocular artifacts (blinks) led to only a modest mean improvement in SNR of 3.6% at the frequencies or interest (see “Supplementary Information 2: Treatment of eye-based artifacts”). Recordings for an individual participant were merged in cases where data were collected across multiple sessions. Trials from the same condition for each participant were then averaged and converted to the frequency domain by fast Fourier transformation (FFT).

Amplitudes for each participant and each color condition were extracted at I_1_, which is an intermodulation component at the sum of the two stimulus frequencies: I_1_ (14.66 Hz) = 1F_A_ (6.66 Hz)+1F_B_ (8 Hz). Amplitudes were also extracted at 1F_B_ (8 Hz), the frequency of the checks of fixed chromaticity. Changes in amplitude with the chromaticity of the F_B_ checks reflect chromatic masking, which has previously been used to infer cortical color tuning functions (Chen & Gegenfurtner, 2021). Amplitudes at 1F_A_ were not extracted because of the fact that both hue angle and hue angle difference (from the hue of the checkerboard checks with fixed chromaticity) vary with condition. Changes in amplitude as a function of color condition could, therefore, be attributable either to changes in chromatic masking with hue angle difference (providing a measure of color tuning as for amplitudes at 1F_B_), or to changes in SSVEP response to particular hues (which do not provide a measure of color tuning), or a combination of these. Because the contributions of these factors would be confounded, color tuning functions cannot be inferred from changes in amplitude with hue angle dissimilarity for 1F_A_.

Consistency in the temporal profiles of our stimuli was ensured by analyzing data from the photodiode which was attached to the display to capture light from both check types within each checkerboard stimulus. Analysis of the stimulus using the photodiode (Fig. 1c) also confirmed that there was no modulation of light in the stimulus at I_1_ (Fig. 1b).

## 3 Experiment 1

In Experiment 1, we sought to measure cortical color tuning functions, and to determine whether there are differences in cortical color tuning functions between “cardinal” color axes aligned with the retinogeniculate color channels L/(L+M) and S/(L+M) and an intermediate color axis. Specifically, if cortical color representations of intermediate colors arise from a combination of activities of two cardinal color mechanisms rather than from activities of an intermediately tuned color mechanism, we would expect “tuning functions” along the intermediate color axis to have a larger bandwidth and smaller peak amplitude than tuning functions for stimuli with equivalent chromatic contrasts along the cardinal axes themselves. Alternatively, if cortical color representations rely on a number of mechanisms tuned to various hues, we would expect the bandwidth of the tuning function for the intermediate axis to be the same as those for the cardinal axes.

### 3.1 Experiment 1: Methods

Eleven participants (four female) completed two experimental sessions, each lasting 2.5 hours, inclusive of cap fitting and comfort breaks. To investigate possible differences in tuning along cardinal and intermediate color axes, three color axes were tested (Fig. 1d): the cardinal L/(L+M) axis in an incremental direction (larger values of L/(L+M) than the white point are reddish), the cardinal S/(L+M) axis in a decremental direction (smaller values of S/(L+M) than the white point are roughly lime green), and an intermediate axis (an orange hue direction). For each axis, the chromaticity of checks flickering at F_B_ was fixed along that axis, and the chromaticity of checks flickering at F_A_ varied on each trial between six hue angles to the fixed axis (0°, 18°, 36°, 54°, 72°, and 90°; Fig. 1d). There were 180 trials in each session (6 hue angle difference conditions x 3 color axes x 10 trials for each combination) presented in a random order, totaling 360 trials across 2 sessions. Checkerboard checks were square and measured 4.8° x 4.8°.

### 3.2 Experiment 1: Results

**Fig. 2.**
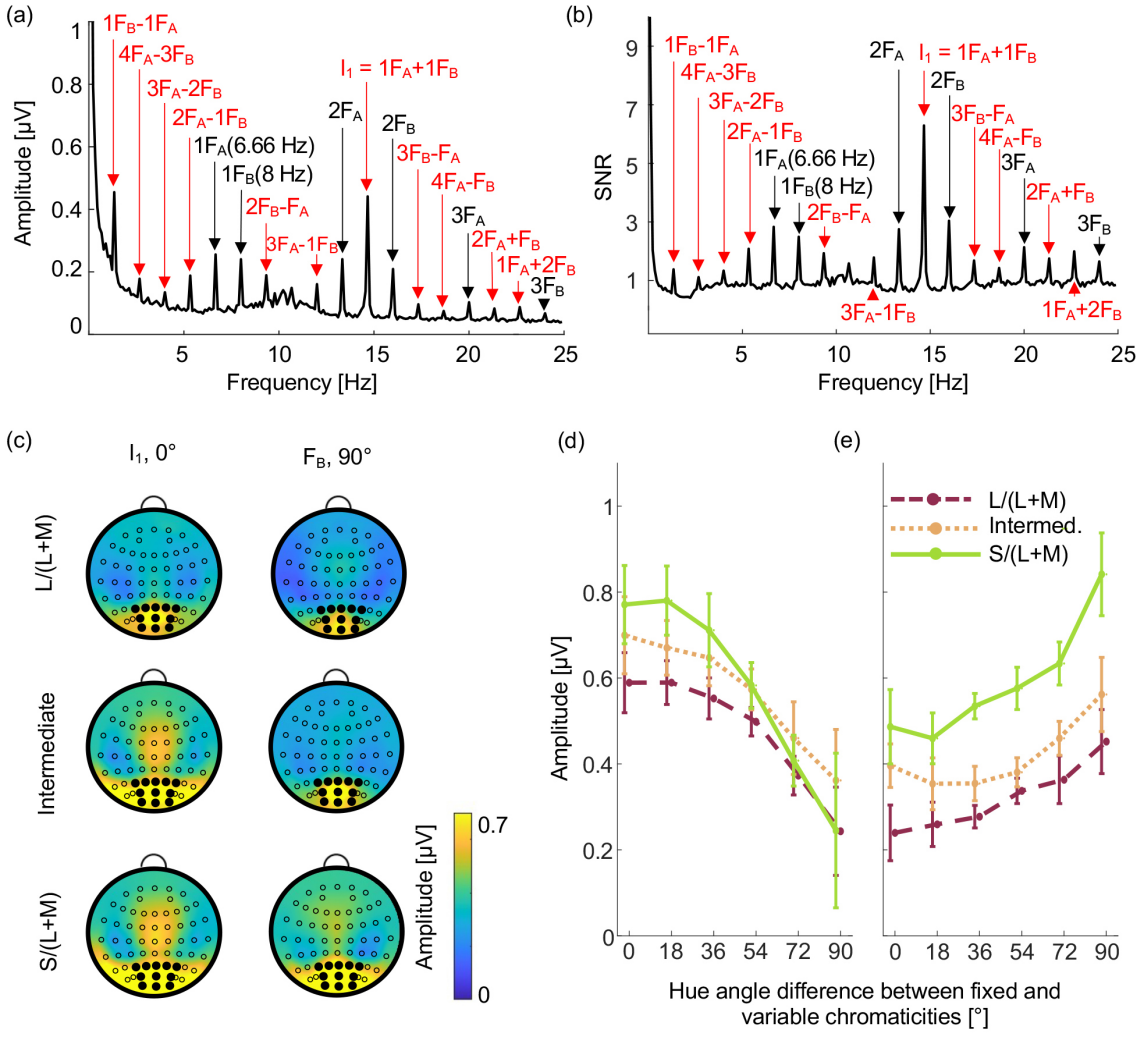
Results of Experiment 1. (a) EEG amplitude as a function of frequency for an example condition (S/(L+M) axis, 0° hue angle difference between check chromaticities), averaged over the 11 participants. Fundamental stimulus frequencies and their harmonics are labeled in black and intermodulation components in red. (b) SNR profile for the same condition. Fundamental stimulus frequencies and their harmonics are labeled in black and intermodulation components in red. (c) Heat maps showing the distribution of signals across electrodes (open circles). Cluster electrode locations are marked by solid black circles. Amplitudes are plotted for the hue angle difference condition that produces the largest amplitudes: The 0° hue angle difference condition for I_1_ (left column) and the 90° hue angle difference condition for 1F_B_ (right column). Heatmaps for all conditions are provided in Supplementary Information 7. (d) Mean amplitude of the intermodulation component I_1_ at 14.66 Hz as a function of the hue angle between the fixed and variable chromaticities for the S/(L+M) axis (green solid line), the intermediate axis (orange dotted line) and the L/(L+M) axis (red dashed line). Error bars are within-participant 95% confidence intervals (O’Brien & Cousineau, 2015). (e) Mean amplitude at the stimulus frequency 1F_B_ (8 Hz) of the fixed chromaticity checks as a function of the hue angle difference between the fixed and variable chromaticities for the S/(L+M) axis (green solid line), the intermediate axis (orange dotted line), and the L/(L+M) axis (red dashed line). Error bars are within-participant 95% confidence intervals.

Analysis in the frequency domain showed strong signals from our cluster electrodes at the two fundamental frequencies, their harmonics (e.g., 2F_A_ and 2F_B_), and at I_1_ (Fig. 2a, b). Across the scalp, peak signals were located centrally and posteriorly (see Fig. 2c for scalp maps). There were additional amplitude peaks from the cluster electrodes at other intermodulation frequencies. We extracted amplitudes at I_1_ and at 1F_B_ for further analyses.

Figure 2 shows the effect of hue dissimilarity between odd and even checks on SSVEP amplitudes at I_1_ (panel d) and 1F_B_ (panel e) for the three color axes. Tuning functions were similar in shape for all three axes, with a broad half-amplitude bandwidth of approximately 90°. Two-way repeated-measures ANOVAs were conducted to test the effect of color axis and hue angle differences on the amplitudes of I_1_ and 1F_B_. The ANOVAs showed that for both I_1_ and 1F_B_, there were significant main effects of hue angle difference (*F*(1.19,11.92) = 21.59, *p* < 0.001, η^2^_p_ = 0.683 for I_1_; *F*(1.84,15.49) = 23.18, *p* < 0.001, η^2^_p_ = 0.699 for 1F_B_). Results from Bonferroni-corrected post hoc paired *t*-tests showed that mean SSVEP amplitudes decreased significantly with hue angle difference for I_1_ and increased significantly with hue angle difference for 1F_B_, indicating significant color tuning (see “Supplementary Information 3: Post-hoc tests for Experiment 1”, Supplementary Table S1 for results based on I_1_, and Supplementary Table S3 for results based on 1F_B_).

There was a significant main effect of color axis for 1F_B_, (*F*(1.14,11.36) = 10.83, *p* = 0.006, η^2^_p_ = 0.520), but not for I_1_ (*F*(1.45,14.51) = 3.17, *p* = 0.084, η^2^_p_ = 0.241). Bonferroni-corrected post hoc paired *t*-tests indicated that there was a significant difference between the L/(L+M) and S/(L+M) axes (*M* = -0.267, *p* < 0.05) for 1F_B_ (see “Supplementary Information 3: Post hoc tests for Experiment 1”, Supplementary Table S2 for results based on I_1_, and Supplementary Table S4 for results based on 1F_B_). A main effect of axis is not unexpected: we roughly equated perceived saturation for the two cardinal axes but there were likely to be residual differences in the neural representation of the saturations of the stimuli along the two axes, which could be eliminated by scaling stimulus saturation differently.

There was no significant interaction between axis and hue angle difference either for I_1_ (*F*(3.11,31.06) = 2.74, *p* = 0.058) or for 1F_B_ (*F*(2.60,26.03) = 2.40, *p* = 0.098). Thus, the effect of hue angle on SSVEP amplitude was consistent across all three axes for both frequencies, implying that tuning functions are shaped similarly for all three axes.

We conducted an additional analysis to determine whether power at fundamental frequencies and their harmonics could explain the shape of the observed tuning function based on signals at I_1_ (Gordon et al., 2019; Zhang et al., 2011). We found that the shape of the tuning function based on I_1_ did not change substantially when plotted as a ratio of power at I_1_ and summed power at the stimulus frequencies and their harmonics. More details on this analysis, and the resulting tuning functions are provided in Supplementary Information 9.

## 4 Experiment 2

The results of Experiment 1 revealed broad color tuning functions with half amplitude bandwidths of about 90° for all three color axes tested. The fact that the tuning function for the intermediate axis had the same bandwidth and a similar peak amplitude to those for the cardinal color axes is compatible with the action of an intermediately tuned color mechanism. In Experiment 2, we sought to investigate whether the color mechanisms we measured in Experiment 1 are spatially selective. Experiment 1’s isoluminant checkerboard stimuli had strong spatial chromatic edges, and thus neural populations responding to strong chromatic edges may underlie the results. To test the effect of chromatic edges on cortical color tuning functions, we reduced the size of the “checks” to single pixels (with edges invisible to participants). If tuning functions change from those measured in Experiment 1 when the checkerboard is constructed using single pixels, then the tuning functions measured in Experiment 1 are attributable to neural populations responsive to chromatic edges (i.e., neurons that are selective for spatial chromatic contrast). If there is no difference between tuning functions measured using a single-pixel checkerboard and those measured in Experiment 1, then the underlying chromatic mechanisms do not rely on chromatic edges. Such mechanisms would rely on neurons with low spatial selectivity, for example, single-opponent neurons or other classes of neurons tuned to low spatial frequencies.

### 4.1 Experiment 2: Methods

**Fig. 3.**
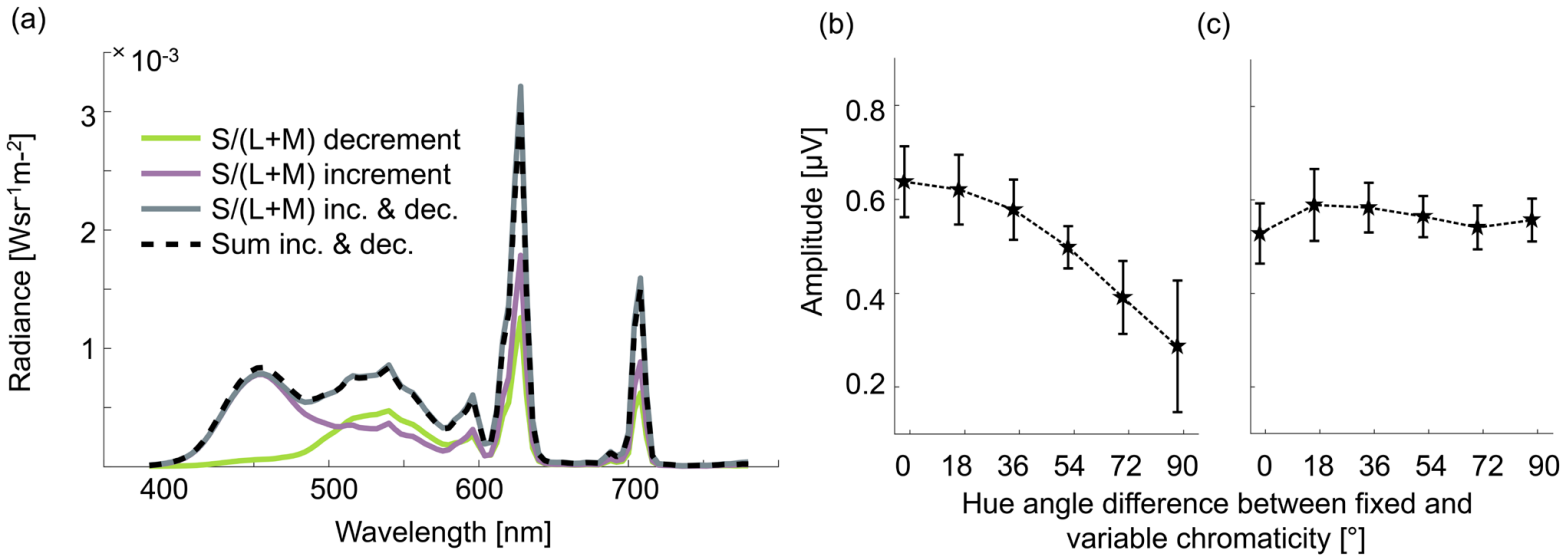
Stimulus calibration check and results for Experiment 2. (a) Light spectra of single-pixel checkerboards. The lime green line shows the spectrum of light from a single-pixel checkerboard with interleaved S/(L+M) decrement and black pixels. The violet line shows the spectrum of light from a single-pixel checkerboard with interleaved S/(L+M) increment and black pixels. The gray solid line shows the spectrum of light from a single-pixel checkerboard with interleaved S/(L+M) decrement and S/(L+M) increment pixels. The black dashed line shows a prediction for the spectrum of the S/(L+M) decrement—S/(L+M) increment single-pixel checkerboard as the sum of the spectra of the two checkerboards with S/(L+M) decrement and S/(L+M) increment pixels separately interleaved with black. (b–c) SSVEP amplitude plotted as a function of hue angle dissimilarity for single-pixel checkerboard stimuli for I_1_ at 14.66 Hz (b) and for 1F_B_ at 8 Hz (c). Error bars are within-participant 95% confidence intervals.

Eleven participants (six female) completed a single 2.5-hour experimental session, inclusive of cap fitting and comfort breaks. We performed a power calculation using G*Power (Faul et al., 2007), which indicated that with three participants, we would have 95% power to detect a main effect of hue angle with the same effect size (Cohen’s *f* = 1.47) as observed for I_1_ in Experiment 1. The sample sizes we chose for Experiments 2–4 were, therefore, conservative: With 11 participants, we expected 95% power to detect a Cohen’s *f* of 0.29. Stimuli consisted of single-pixel checkerboards with checks measuring 0.096° x 0.096°, which otherwise had the same spatial organization as the checkerboard depicted in Figure 1a. Edges between individual checks were perceptually indistinguishable to participants. The chromaticity of checks flickering at F_B_ was fixed, and the chromaticity of checks flickering at F_A_ varied between six hue angles to the fixed chromaticity (0°, 18°, 36°, 54°, 72°, and 90°). There were 120 experimental trials (20 for each condition), where hue conditions were presented in a random order. Since the S/(L+M) axis produced somewhat larger amplitudes in Experiment 1 than the other two axes, Experiment 2 probed the S/(L+M) axis only. Color calibration was checked for single-pixel checkerboards using a SpectraScan PR655 spectroradiometer (PhotoResearch Chatsworth, CA, USA). We found that the spectrum of light from a composite checkerboard was predictable as the linear sum of light from odd and even checks presented separately, showing that light is displayed independently in odd and even checks (Fig. 3a).

### 4.2 Experiment 2: Results

Figure 3 shows the amplitudes of I_1_ (Fig. 3b) and 1F_B_ (Fig. 3c) against the difference in hue angle between odd and even single-pixel checks. One-way repeated-measures ANOVAs examined the effect of hue angle difference on the amplitudes of I_1_ and 1F_B_. There was a significant effect of hue angle difference on amplitude for I_1_ (*F*(1.33,13.26) = 10.32, *p* = 0.004, η^2^_p_ = 0.508), but not for 1F_B_ (*F*(2.74,27.44) = 0.675, *p* = 0.562, η^2^_p_ = 0.063). Results from post hoc paired *t*-tests indicated that mean amplitude decreased with hue angle difference for I_1_, but did not change with hue angle difference for 1F_B_ (see “Supplementary Information 4: Post hoc tests for Experiment 2”, Supplementary Table S5 for results based on I_1_, and Supplementary Table S6 for results based on 1F_B_). In an additional analysis, we found that the shape of the tuning function based on SSVEPs at I_1_ could not be explained by summed power at the stimulus frequencies and their harmonics (Supplementary Information 9).

## 5 Experiment 3

The results of Experiment 2 showed that color tuning functions based on I_1_ for stimuli without perceptible spatial chromatic edges did not differ from those observed in Experiment 1 for stimuli with strong chromatic edges. This implies that the color mechanism(s) revealed by I_1_ are not spatially selective or are tuned to very low spatial frequencies. However, for 1F_B_ we observed a difference in the shape of the tuning function between the 4.8° checks presented in Experiment 1 and the invisible 0.096° checks presented in Experiment 2, implying that the color mechanism(s) revealed by 1F_B_ are spatially selective. To further investigate the effect of chromatic edges on tuning functions derived from signals at 1F_B_, in Experiment 3 we systematically manipulated check sizes. We measured tuning functions at 1F_B_ and at I_1_ at four check sizes, varying between the check sizes used in Experiments 1 and 2. Because we found no difference between tuning functions based on I_1_ between Experiments 1 and 2, we expected to find no effect of check size on amplitudes at I_1_. We expected an effect of check size on amplitudes at 1F_B_, with flat tuning functions for the single-pixel checkerboard and steeper tuning functions for checkerboards with larger check sizes.

### 5.1 Experiment 3: Methods

Eleven participants (five female) completed two 2.5-hour experimental sessions, inclusive of cap fitting and all breaks. There were four check size conditions: 0.096° (as in Experiment 2), 0.4°, 1.3°, and 4.8° (as in Experiment 1). To limit the duration of testing sessions, there were four hue angle difference conditions rather than the six used in Experiments 1 and 2. These were 0°, 30°, 60°, and 90°. Checks flickering at F_B_ (8 Hz) at a fixed chromaticity modulated between equal energy white and a decrement along S/(L+M) axis (upper panel of Fig. 1d). Two testing sessions were conducted, separated by at least 1 day. There were 160 experimental trials per session (10 trials for each combination of check size and hue angle difference), totaling 320 trials (20 trials in total for each stimulus type).

### 5.2 Experiment 3: Results

**Fig. 4.**
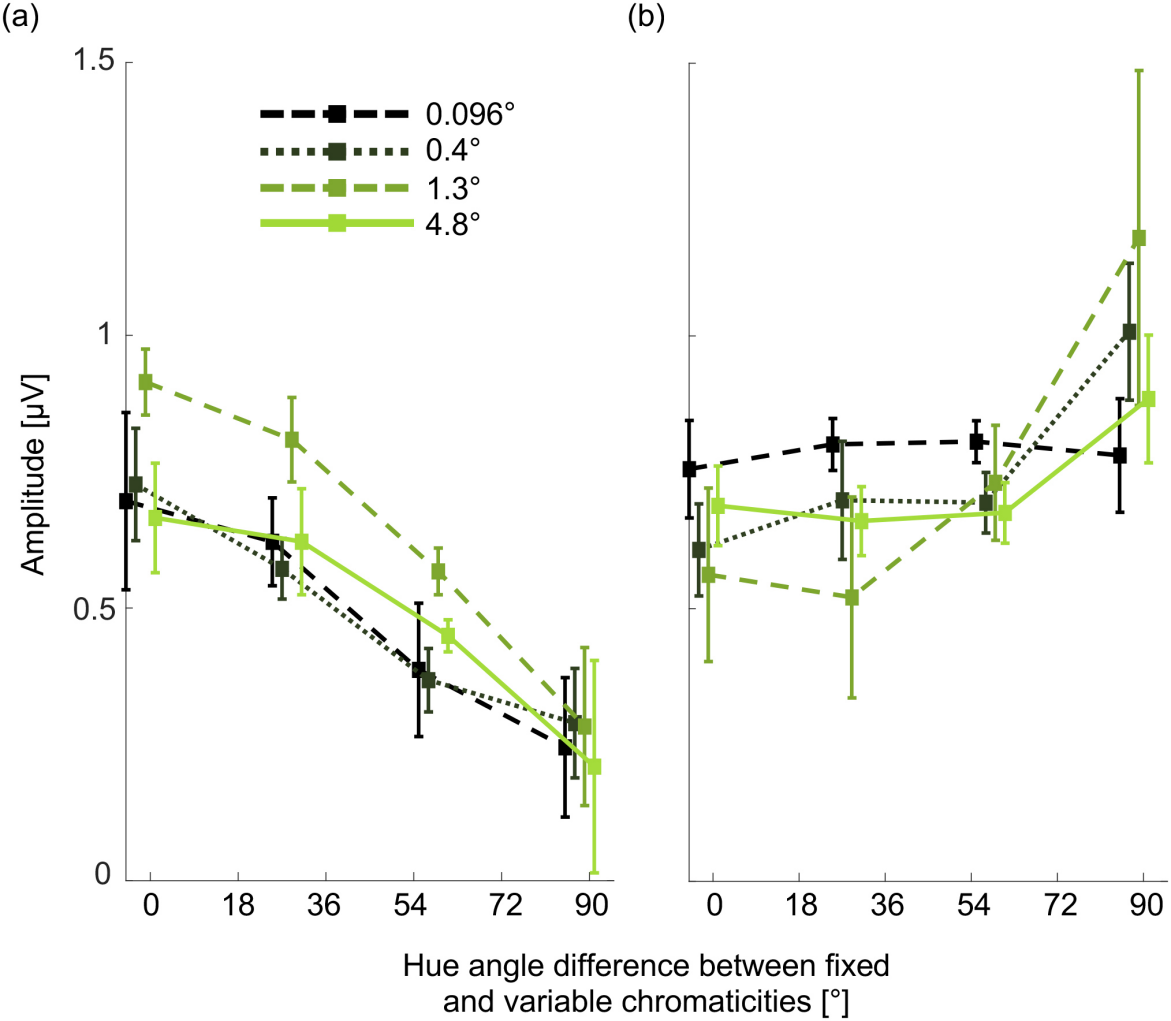
Results of Experiment 3. SSVEP amplitudes are plotted as a function of hue angle dissimilarity (x-axis) and check size (legend). (a) For I_1_ at 14.66 Hz. (b) For 1F_B_ at 8 Hz. Error bars are within-participant 95% confidence intervals.

Figure 4 shows the effect of check size and hue angle difference on amplitudes at I_1_ (panel a) and at 1F_B_ (panel b). The figure shows that for I_1_, check size does not affect the shape of the tuning functions observed. However, for 1F_B_, in agreement with the results of Experiment 2, there is no tuning for the single-pixel checkerboard, but similar tuning functions as observed in the results of Experiment 1 for the larger check sizes.

We conducted two-way repeated-measures ANOVAs to test the effect of hue angle difference and check size on amplitudes at I_1_ and 1F_B_. There were significant main effects of check size and hue angle on signal amplitude for I_1_ (*F*(2.51,25.11) = 7.79, *p* = 0.001, η^2^_p_ = 0.438 for check size; *F*(1.11,11.11) = 26.80, *p* < 0.001, η^2^_p_ = 0.728 for hue angle difference), but no significant interaction (*F*(2.68,26.77) = 1.91, *p* = 0.157, η^2^_p_ = 0.160). Post hoc paired *t*-tests with Bonferroni correction indicated that mean amplitudes for 1.3° checks were somewhat stronger than for other check size conditions regardless of hue angle (see “Supplementary Information 5: Post-hoc tests for Experiment 3”, Supplementary Table S7), and that mean amplitudes decreased with hue angle for I_1_, regardless of check size (see “Supplementary Information 5: Post-hoc tests for Experiment 3”, Supplementary Table S8). For 1F_B_ there was a significant main effect of hue angle difference (*F*(1.38,13.79) = 14.79, *p* < 0.001, η^2^_p_ = 0.597), but there was no significant main effect of check size (*F*(1.37,13.71) = 0.378, *p* = 0.614, η^2^_p_ = 0.036; see Supplementary Table S9 for results of post hoc paired *t*-tests). Results from post hoc paired *t*-tests with Bonferroni correction indicated that mean amplitudes increased with hue angle difference (see “Supplementary Information 5: Post hoc tests for Experiment 3”, Supplementary Table S10). There was a significant interaction between check size and hue angle difference for 1F_B_ (*F*(1.86,18.57) = 4.76, *p* = 0.023, η^2^_p_ = 0.322).

In summary, the results of Experiment 3 show that the shapes of tuning functions observed at I_1_ are independent of check size, confirming the results of Experiment 2. This is also the case for tuning functions based on the ratio of power at I_1_ and summed power at the stimulus frequencies and their harmonics (Supplementary Information 9). In contrast, the shapes of tuning functions observed at 1F_B_ do vary with check size: chromatic tuning is present for larger checks but is lost for single-pixel (imperceptible) checks, again confirming the results of Experiment 2. The difference between the effects of check size on tuning functions at I_1_ and at 1F_B_ implies that the two frequencies reveal the actions of different cortical color mechanisms. Signals at I_1_, not reliant on chromatic edges, likely originate from populations of neurons with low spatial selectivity, while signals at 1F_B_, reliant on chromatic edges, likely originate from spatially selective neurons (see Discussion section for further interpretation of this result).

## 6 Experiment 4

The tuning functions observed in the results of Experiments 1–3 were broadband with half-amplitude bandwidths of about 90°, but did not reach an asymptote at the largest hue angle differences we measured of 90°. In Experiment 4, we aimed to measure a tuning function for a cortical color mechanism around the entire hue circle. We kept the fixed chromaticity checks along the S/(L+M) axis in a decremental direction, but varied the variable chromaticity checks around the full hue circle in 12 steps. If the underlying color mechanisms are broadly tuned and unipolar (representing color differences between gray and a preferred hue), we would expect to see a further reduction in amplitudes at I_1_ beyond 90° hue angle dissimilarity. Alternatively, if the underlying color mechanisms are bipolar, we would expect to see a second peak in amplitudes at I_1_ centered on 180° hue angle dissimilarity.

### 6.1 Experiment 4: Methods

Ten participants (seven female, three authors) completed the main part of Experiment 4 in one experimental session lasting about 3 hours, inclusive of capping and all breaks. Stimuli were isoluminant flickering checkerboards with a check size of 1.3° (which elicited the largest signals in Experiment 3). The fixed chromaticity flickering at F_B_ (8 Hz) was a decrement on the S/(L+M) axis (indicated by the solid line in the upper panel of Fig. 1d). The chromaticity of the variable checks flickering at F_A_ (6.66 Hz) spanned the full hue circle in steps of 30° (hue angle differences ranged from 0° to 330°). Each of the 12 hue angle conditions was presented 20 times, totaling 240 trials. SSVEP amplitudes were extracted at I_1_ and at 1F_B_.

To investigate the generalizability of tuning functions between color axes, we also measured full tuning functions with a check size of 1.3° centered on three further color axes in addition to the S/(L+M) decrement axis: the L/(L+M) increment axis, and two intermediate axes—the positive diagonal in the MacLeod and Boynton (1979) chromaticity diagram in a purple direction and the negative diagonal in a blue direction (Fig. 6a). We conducted these measurements with a smaller sample of 5 participants (aged 19–30 years, *M* = 24, 1 author), since modeling the effect of a smaller sample size on the results from 10 participants for the S/(L+M) decrement axis found that clear tuning functions could be expected with the reduced sample.

In Experiments 2 and 3, we found differences between color tuning functions derived from I_1_ versus 1F_B_ dependent on checkerboard check sizes. To investigate whether these differences persist for tuning functions around the full hue circle we measured, in a sample of five participants (aged 22–30 years, *M* = 27, 1 author), tuning functions around the full hue circle for the S/(L+M) decrement axis both for single-pixel checkerboards and for checkerboards with 1.3° checks.

### 6.2 Experiment 4: Results

**Fig. 5.**
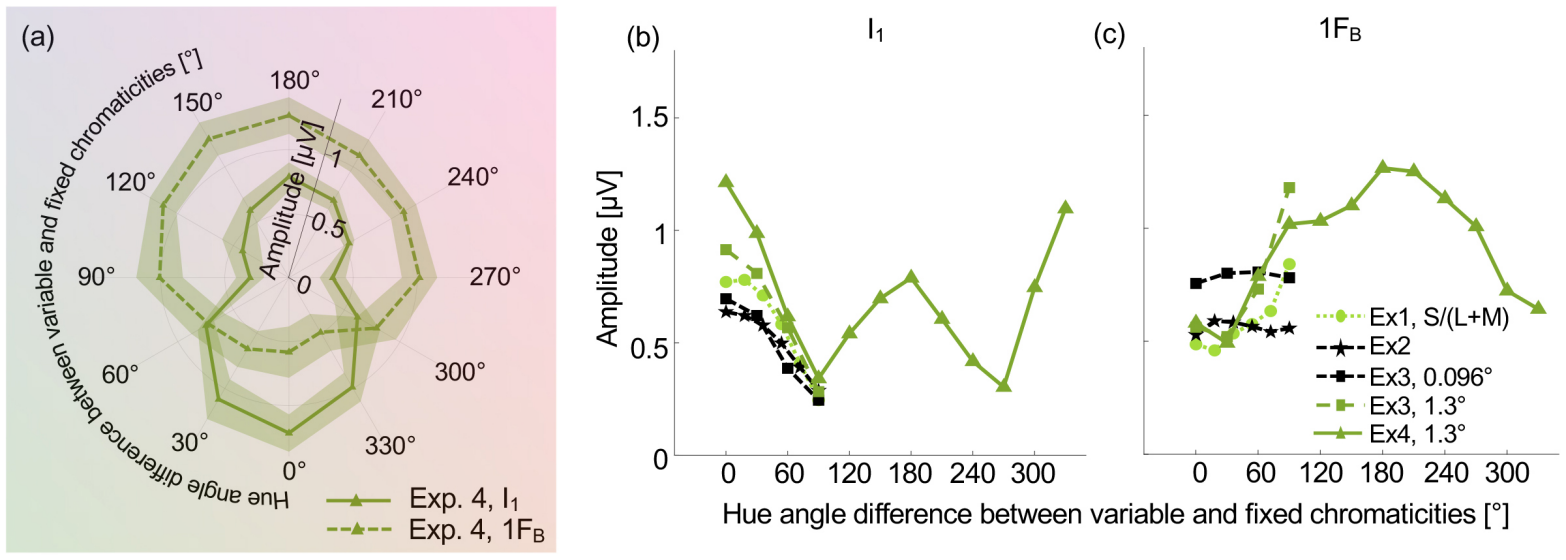
Results of the main part of Experiment 4. (a) Polar tuning functions plotted at I_1_ (solid line) and 1F_B_ (dashed line), where the fixed chromaticity was an S/(L+M) decrement (180°), and the variable chromaticity progressed around the full hue circle. Error envelopes are within-participant 95% confidence intervals. The colored background provides an indication of the variable chromaticity at each hue angle, but is not color calibrated. (b) Tuning functions for decrements in S/(L+M) comparing the results of Experiment 4 with those from Experiments 1–3 for I_1_ (14.66 Hz). (c) Tuning functions for decrements in S/(L+M) comparing the results of Experiment 4 with those from Experiments 1–3 for 1F_B_ (8 Hz). For Experiment 3, results are shown in panels (b) and (c) only for the 0.096° and 1.3° check size conditions.

Main results for the S/(L+M) decrement axis are presented in Figure 5. There is a peak in the tuning function derived from I_1_ centered at a 0° hue angle difference, as expected from the results of Experiments 1–3. However, there is a second smaller peak centered at a hue angle difference of 180° when the variable chromaticity (an S-cone increment) is opposite to the fixed chromaticity (an S-cone decrement). For 1F_B_ there is a minimum at a hue angle difference of 0° as expected from the results of Experiments 1 and 3. There is no evidence of a second minimum at a hue angle difference of 180°. The shapes of the tuning functions are highly reliable across split halves of the data (see “Supplementary Information 8: Split-half reliability of tuning functions from Experiment 4”. For a more detailed analysis, including the effect of trial numbers on reliability, see Rozman et al. (2025)). One-way repeated measures ANOVAs were used to investigate the effect of hue angle difference on amplitudes at I_1_ and at 1F_B_. There was a significant effect of hue angle difference on amplitudes at I_1_ (*F*(2.131,19.18) = 18.23, *p* < 0.001, η^2^_p_ = 0.677) and at 1F_B_ (*F*(2.289,30.15) = 11.10, *p* < 0.001, η^2^_p_ = 0.552). Post hoc paired *t*-tests showed that amplitudes at I_1_ for a 0° hue angle difference were significantly greater than amplitudes at all other angles, except for 30° and 330°. The amplitude for a hue angle difference of 180° was not significantly different from those of its most proximal hue angle difference conditions, 150° and 210° (see Supplementary Information 6 “Post hoc tests for Experiment 4”, Supplementary Table S11 for results based on I_1_ and Supplementary Table S12 for results based on 1F_B_). The shape of the tuning function was preserved when the potential influence of power at the stimulus frequencies was accounted for by plotting the ratio of power at I_1_ and summed power at the stimulus frequencies and their harmonics (Supplementary Information 9). Scalp heatmaps for all conditions are provided in Supplementary Information 7.

**Fig. 6.**
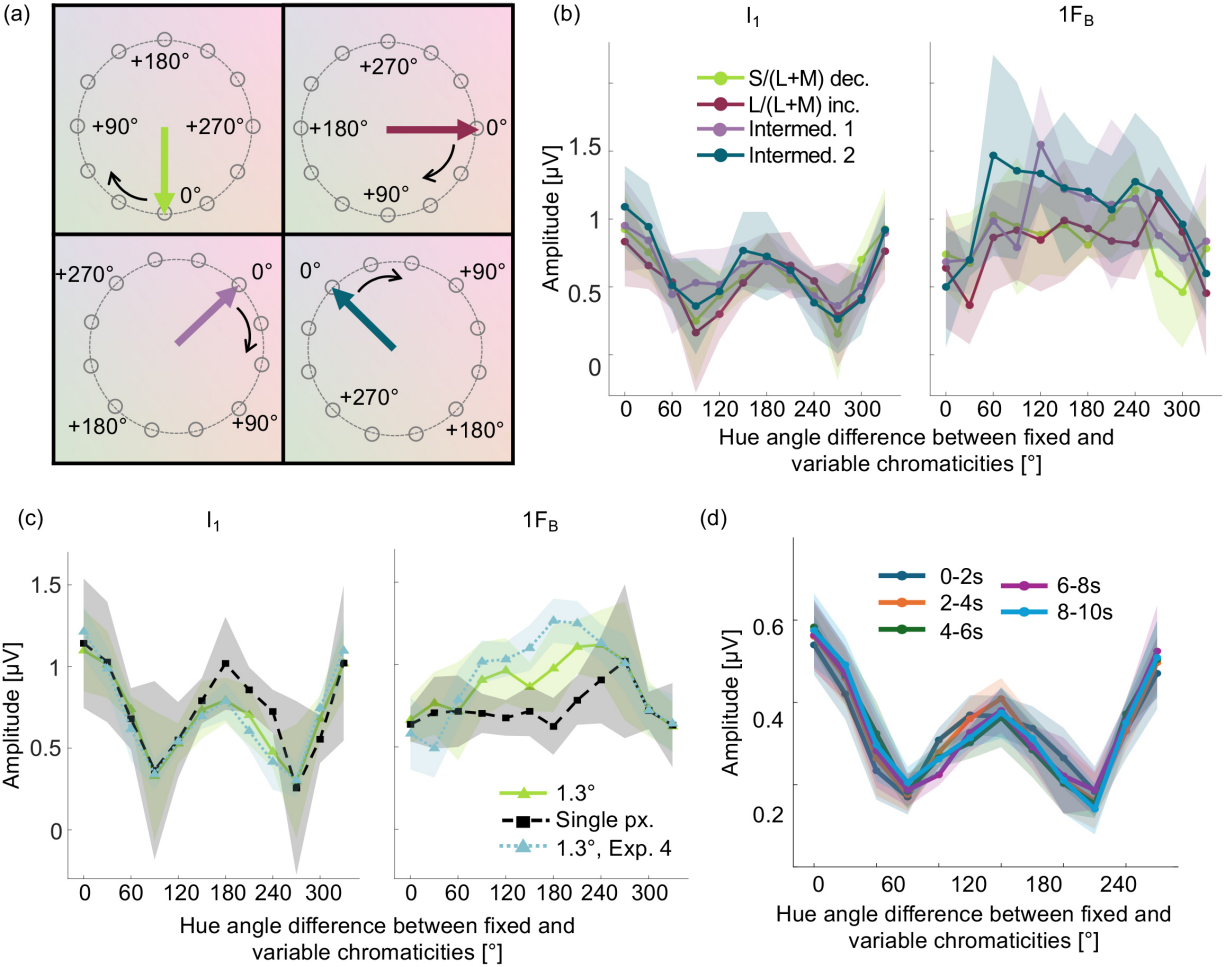
Full 360° color tuning functions for additional color axes and for single-pixel checkerboards. (a) the four axes tested plotted in a polar version of the MacLeod–Boynton chromaticity diagram, showing the hue angle difference conditions for each axis. For each sub-panel, the color axis tested is indicated by the solid arrow. Colors are symbolic. (b) Color tuning functions derived from I_1_ (left panel) and from 1F_B_ (right panel), for the four color axes shown in panel (a). For each tuning function, the error cloud indicates 95% confidence intervals. (c) Color tuning functions around the full hue circle derived from I_1_ (left panel) and from 1F_B_ (right panel) for the S/(L+M) decrement axis for single-pixel checkerboards (dashed black) and for checkerboards with 1.3° checks (full green). Tuning functions from the main part of Experiment 4 (plotted in Fig. 5) for checkerboards with 1.3° checks are indicated by the dotted blue lines for comparison. Error clouds indicate within-participant 95% confidence intervals. (d) Color tuning functions based on I_1_ for 5 subsequent 2-second epochs within the 10-second trials. Error clouds show within-participant 95% confidence intervals.

Results of our measurements of tuning functions for further color axes in five participants showed that tuning functions derived from I_1_ had the same shape as that measured for the S-cone decrement axis plotted in Figure 5b, with a peak at a 0° hue angle difference from the chromaticity of the fixed checks, and a second peak at a 180° hue angle difference (Fig. 6b, left panel). A two-way repeated measures ANOVA with factors for axis and hue angle found no significant main effect of axis (*F*(1.52,6.08) = 1.089, *p* = 0.373, η^2^_p_ = 0.214) and no significant interaction between hue axis and hue angle difference (*F*(2.87,11.47) = 1.48, *p* = 0.271, *η*_p_^2^ = 0.271). For tuning functions derived from 1F_B_, the three additional axes had the same shape as that for the S-cone decrement axis plotted in Figure 5c, with a single peak at a 180° hue angle difference (Fig. 6b, right panel).

In agreement with the results of Experiments 2 and 3, we found that the color tuning function derived from I_1_ does not depend on check size. For single-pixel checkerboards, we found that the tuning function has the same bipolar shape as for checkerboards with 1.3° checks (Fig. 6c, left panel). Also in agreement with the results of Experiments 2 and 3, we found that the tuning function derived from 1F_B_ does vary with check size. This tuning function has a single central peak at a hue angle difference of 180° for checkerboards with 1.3° checks, but no consistent relationship with hue angle difference for single-pixel checkerboards (Fig. 6c, right panel).

To explore the potential impact of adaptation on the tuning functions we observe, we plotted separate tuning functions based on I_1_ for 5 independent consecutive 2-second epochs within each 10.5-second trial, beginning at the trial onset (Fig. 6d). If adaptation influences the tuning functions, we should expect to see changes in tuning functions between the first epoch in trials where adaptation should be lowest (0–2 seconds) and the last epoch in trials where adaptation should be greatest (8–10 seconds). However, a two-way repeated measures ANOVA revealed no significant main effect of epoch (*F*(1.45,13.06) = 0.025, *p* = 0.94, η^2^_p_ = 0.003) and no significant interaction between epoch and hue angle difference (*F*(4.92,44.25) = 2.07, *p* = 0.089, η^2^_p_ = 0.187), indicating no effect of adaptation on the shape of tuning functions. There was a significant main effect of hue angle difference (*F*(2.21,19.86) = 18.66, *p* < 0.001, η^2^_p_ = 0.675) as in the main analysis over 9-second epochs within trials.

In summary, tuning functions revealed by amplitudes at I_1_ showed a second peak at a 180° hue angle difference to the chromaticity of the fixed checks, but there was no second peak in the tuning functions extracted from 1F_B_. This difference in results between I_1_ and 1F_B_ again implies that they arise from different underlying neural populations. The presence of two peaks in the tuning functions at I_1_ is consistent with a bipolar color representation.

## 7 Analyses of Additional Intermodulation Components

**Fig. 7.**
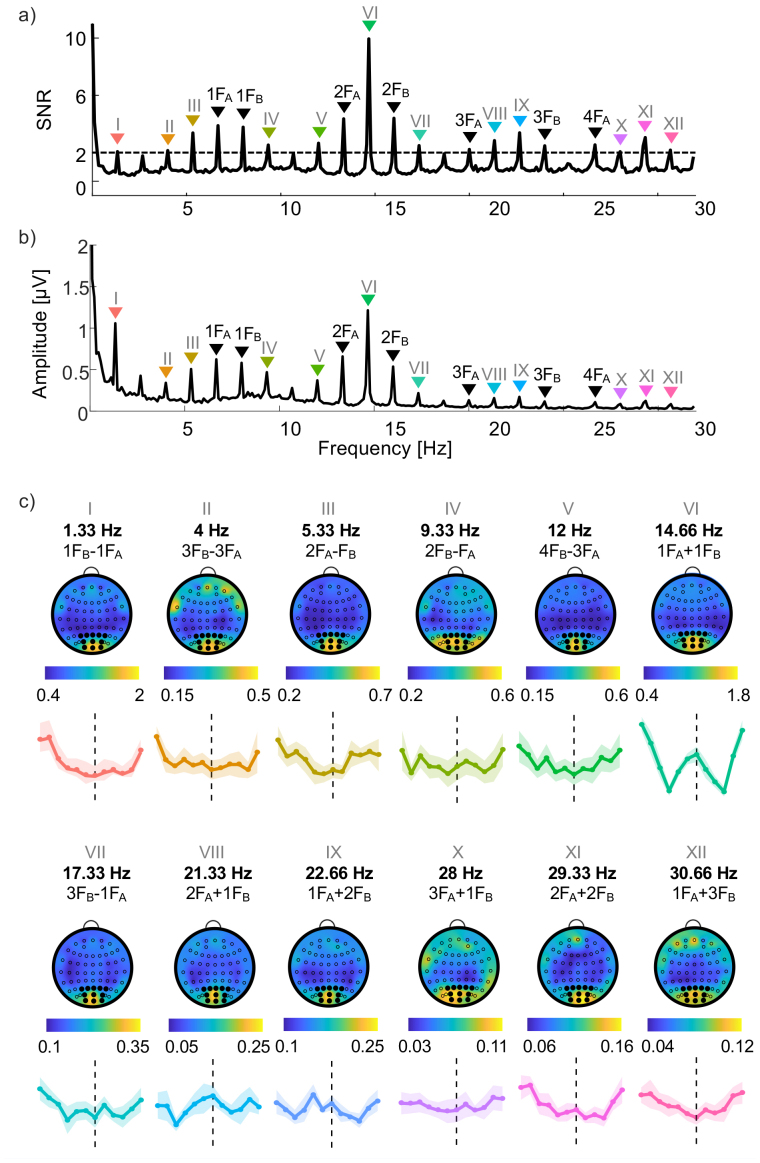
Signal heatmaps and tuning functions based on intermodulation components surpassing 2 SNR in Experiment 4. (a) SNR plotted as a function of frequency in Hz. Signal frequencies and their harmonics are labeled in black, and intermodulation components are labeled with unique Roman numerals and with colored inverted triangles (note that the colors are not related to hue angles tested, but are chosen to provide visual labels for the different intermodulation components). (b) The equivalent plot as in panel (a) but showing amplitude as a function of frequency in Hz. (c) Individual panels showing normalized tuning functions for all the intermodulation components exceeding 2 SNR, scaled to match in area under the curve. These tuning functions are colored to match the arrows of the corresponding intermodulation components in panels (a) and (b), and are also labeled with the unique Roman numerals. Error clouds indicate within-participant 95% confidence intervals. Above the tuning function for each intermodulation component is a normalized scaled scalp electrode heatmap showing the amplitudes of that intermodulation component across electrodes for the 0° hue angle difference condition. Cluster electrodes are indicated by filled black circles and other electrodes by open circles.

It is thought that different intermodulation components may arise from different subpopulations of neurons (Appelbaum et al., 2008; Gordon et al., 2019; Regan, 1966). For example, the subpopulation of neurons underlying the difference between F_A_ and F_B_ might be separate from the population underlying the sum of F_A_ and F_B_, even though both intermodulation components arise from interactions between responses at the stimulus frequencies (at 1F_A_ and 1F_B_). These potentially different populations may also demonstrate distinct color tuning and may be differently spatially distributed across the cortex. We, therefore, conducted additional analyses of the data from 10 participants for the S/(L+M) decrement axis in Experiment 4 to plot tuning functions for intermodulation components other than I_1_, and to visualize their locations in cortical electrode maps. Firstly, we extracted SNR profiles for a grand mean across participants in the 0° hue difference condition (where intermodulation components are expected to be greatest). Intermodulation components (i.e., those where the frequency peak is composed of either an addition or subtraction of multiples of F_A_ and F_B_) were extracted when the SNR peak surpassed a criterion of 2 (Fig. 7a). This procedure identified 12 qualifying components (Fig. 7a, b). One response at 4 Hz (labeled orange in Fig. 7) could either be an intermodulation component (3F_B_-3F_A_) or a subharmonic of F_B_ (F_B_/2). Since we found no peak at the equivalent subharmonic of F_A_, this peak is more likely to be an intermodulation component. Tuning functions were calculated for the channels in the analysis cluster and visualized using scaled line plots. Tuning functions for different intermodulation components had different shapes (e.g., compare tuning functions for 1F_A_+1F_B_ (VI) and for 2F_A_+2F_B_ (XI) in Fig. 7c).

Mean (across participants) spatial signal distributions were visualized using amplitude scalp heatmaps, using a relativized scale between frequencies so that the maximum values are always plotted in yellow and minimum values in dark blue (Fig. 7c). Comparison of the cortical surface heatmaps for different intermodulation components revealed that their locations are broadly consistent. Most intermodulation components are restricted to occipital electrodes, but there is a hint that some components may be more broadly distributed (e.g., 9.33 Hz (IV) and 28 Hz (X)). However, we found some substantial differences in tuning functions for different intermodulation components. In results for Experiment 4, some functions show a *reduction* in amplitude for the opponent hue (e.g., 30.66 Hz (XII)), in contrast to the characteristic peak observed in I_1_. At the 21.33 Hz (VIII) intermodulation component, tuning appears to be reversed, with a higher relative peak for the opponent than for the proximal hues (Fig. 7c). Overall, peaks are less pronounced for intermodulation components other than I_1_, but nonetheless may reveal different color tuning properties for different neural subpopulations.

## 8 Discussion

Our results show that human cortical color tuning functions can be effectively measured by intermodulation of SSVEPs. We separately frequency tagged two colors spatially interleaved in checkerboards, and analyzed signals at an intermodulation frequency (I_1_). As we predicted, I_1_ amplitudes systematically reduced with hue angle dissimilarity. Resulting tuning functions were broad, with half-amplitude bandwidths of about 90° in hue angle, consistent with existing reported bandwidths of color tuning functions measured psychophysically (Lindsey & Brown, 2004; Sankeralli & Mullen, 1997) and electrophysiologically (e.g., Wachtler et al., 2003). Since stimuli and the color spaces used to define them have varied between existing studies, a precise quantitative comparison between bandwidths of tuning functions derived from different methods would require measurements of tuning functions using the same stimuli. The results of Experiment 1 showed similar tuning functions for cardinal and intermediate color axes. The results of Experiments 2 and 3 showed that the shape of the tuning functions extracted from I_1_ did not depend on the presence of perceptible chromatic edges or on the sizes of checkerboard checks. The results of Experiment 4 showed that tuning functions around the whole hue circle exhibit a bipolar shape with similar 90° half-amplitude bandwidths at both peaks. We interpret each of these findings in turn.

To assess the independence or interaction between color representations along “cardinal” retinogeniculate color axes and intermediate color axes, in Experiment 1 we measured tuning functions centered on both cardinal axes and on an intermediate axis. If colored stimuli specified along an intermediate axis are represented by a combination of activities in the two cardinal mechanisms, then observed “tuning functions” along the intermediate axis should have a larger bandwidth and a smaller peak amplitude than tuning functions for stimuli with equivalent contrast along the cardinal axes. However, we found very similar peak amplitudes and bandwidths for tuning functions centered along an intermediate axis as for the cardinal axes, implying the action of an independent intermediately tuned cortical color mechanism. This finding is in agreement with existing results from psychophysics (Gegenfurtner & Kiper, 1992; Hansen & Gegenfurtner, 2013; Krauskopf & Gegenfurtner, 1992; Krauskopf & Zaidi, 1986; Webster & Mollon, 1991), fMRI (Goddard et al., 2010; Kuriki et al., 2015), and from Chen and Gegenfurtner’s (2021) SSVEP study.

How are inputs from the cardinal retinogeniculate color mechanisms combined in the cortex to produce intermediately tuned color mechanisms? Gegenfurtner and colleagues have suggested that broad tuning with a half-amplitude bandwidth greater than 30° typically results from a linear combination of cone-opponent signals (Chen & Gegenfurtner, 2021; Hansen & Gegenfurtner, 2006). However, nonlinear combinations of cone-opponent signals could also result in broad tuning functions along intermediate axes depending on how the inputs are combined. In their “hue sweep” SSVEP study, Kaneko et al. (2020) found that SSVEP amplitudes in response to colors along intermediate axes could be greater than amplitudes in response to colors along cardinal axes, which they interpreted as evidence that intermediately tuned color mechanisms result from nonlinear combinations of the cardinal mechanisms. In our Experiment 1 results, the amplitudes of I_1_ along the intermediate axis were approximately midway between those of the two cardinal axes. This pattern, according to Kaneko et al., is in favor of a nonlinear combination of cardinal mechanisms. But the results of both studies could also be explained by linear combination followed by independent gain control for each second stage color mechanism.

To differentiate the contributions of spatially selective and non-selective neurons to the tuning functions measured in Experiment 1, in Experiments 2 and 3 we manipulated chromatic edges in the stimuli. We found no effect of check size on the bandwidth of tuning functions based on I_1_, surprisingly, even when the stimuli lacked perceptible chromatic edges. Check sizes of 1.3° generated slightly higher amplitudes than the other check sizes, but no significant interaction between check size and hue angle dissimilarity suggested that the larger amplitudes could result from responses by other spatial frequency-tuned neural populations that are not hue specific. In contrast to tuning functions based on our main target I_1_, tuning functions based on 1F_B_ did depend on chromatic edges. For the larger checkerboard checks (similar to those used by Chen & Gegenfurtner, 2021), tuning functions derived from 1F_B_ were roughly the inverse of those derived from I_1_, implying the action of the same chromatic mechanisms. However, for our single-pixel checkerboard stimuli, tuning functions derived from I_1_ matched those measured using larger check sizes, but tuning functions derived from 1F_B_ were flat. The color mechanism revealed by signals at 1F_B_ is, therefore, sensitive to spatial chromatic edges, but that revealed by signals at I_1_ is not. We note that our stimulus was parafoveal and peripheral and did not include the central 5.7° of the visual field (to avoid the macular pigment). It is possible that chromatic edges may influence signals at I_1_ for foveal stimuli, but not for parafoveal/peripheral stimuli. Future studies could investigate foveal stimuli, though care must be taken to ensure stimulus isoluminance as macular pigment density varies with eccentricity and between observers.

Surprisingly, for tuning functions based on I_1_ measured around the whole hue circle, we found a second peak at a hue angle difference of 180° that had about half the amplitude of the primary peak centered at a hue angle difference of 0°. We did not observe this pattern for tuning functions based on 1F_B_, where there was a single minimum at a hue angle difference of 0° and a single maximum at a hue angle difference of 180°. What could explain this pattern of findings? We assume, as argued by Chen and Gegenfurtner (2021), that tuning functions plotted by extracting 1F_B_ are based on chromatic masking. Is chromatic masking more effective for colors 180° distinct from the target than for colors 90° distinct from the target? We have not identified any psychophysical data that could address this, but it seems unlikely: masking should be minimal at 90° when mask and target chromaticities are carried by orthogonally tuned color mechanisms. However, target signals will also be influenced by simultaneous contrast (Ekroll & Faul, 2012), which will enhance color signals, maximally when surrounding checks have the complementary chromaticity, but will have no effect when the surround checks have an orthogonal chromaticity. The single peak in tuning at 1F_B_ could, therefore, be explained by a combination of masking and simultaneous contrast.

We found second peaks in tuning functions derived from I_1_ at colors complementary to the fixed colors, for the S-decrement axis, and for three additional color axes. We also found the same bipolar-shaped tuning function for single-pixel checkerboards as for checkerboards with 1.3° checks. The second peaks in amplitude at hue angle differences of 180° must arise when the same neural populations are driven by both checkerboard check colors. One possible explanation for these results is the action of bipolar color mechanisms (Chatterjee & Callaway, 2003; Conway et al., 2002) that respond to both checkerboard check colors when they are complementary. Alternatively, intermodulation could arise within rectified ON and OFF channels, consistent with multiple unipolar channels for color in the cortex (Emery et al., 2017, 2023; Malkoc et al., 2005). For example, S-cone OFF channels would respond to the onsets of S-cone decrement checks (at F_A_) and to the offsets of S-cone increment checks (at F_B_), and S-cone ON channels would show the opposite pattern. Intermodulations could thus occur separately within ON and OFF channels, but these separate responses would be at the same intermodulation frequency and not separable in our results. Further work could attempt to measure responses in ON and OFF channels separately by manipulating the form of the temporal waveform to preferentially stimulate either ON or OFF mechanisms (Krauskopf & Zaidi, 1986; Stockman et al., 2017).

In the results of an additional exploratory analysis on consecutive sub-epochs within the 10.5-second trials of Experiment 4, we found no significant effects of epoch, implying that adaptation (assumed to be lower at the beginning than at the end of trials) does not cause measurable changes in the tuning functions. A recent study by Zhang et al. (2023) found only modest adaptation of SSVEPs to chromatic gratings with a relatively slow time course (a half-life of 20 seconds). Over our short (by the standards of studies using SSVEPs) 10.5-second trials, Zhang et al.’s findings suggest that we should not expect much adaptation of SSVEP amplitudes. Nonetheless, the results of our sub-epoch analysis imply that adaptation is not responsible for the bipolar profiles of the tuning functions we have measured.

SSVEP responses reflect activity from multiple neural populations. However, it may be possible to separate the activities of different neural populations by investigating different intermodulation components (Alp et al., 2016; Appelbaum et al., 2008; Gordon et al., 2019; Regan, 1966). We plotted tuning functions for all the intermodulation components that met a criterion of 2 SNR in Experiment 4 (Fig. 7a). By eye, we found that tuning functions based on some intermodulation components showed a second peak at a 180° hue angle difference as for I_1_ (3F_B_-1F_A_, 2F_A_+1F_B_ and 1F_A_+2F_B_), while tuning functions based on others did not have the second peak (1F_B_-1F_A_, 3F_B_-3F_A_, 4F_B_-3F_A_, 2F_A_+2F_B_ and 1F_A_+3F_B_). It is possible that these different groups of intermodulation components reflect the actions of different neural populations with different color tuning properties. However, the scalp map distributions of the different components are not obviously different (Fig. 7).

When SSVEP responses at intermodulation frequencies are compared across stimulus conditions, it is important to consider SSVEP responses at the stimulus frequencies and their harmonics (Gordon et al., 2019; Zhang et al., 2011), which may also change across stimulus conditions. Comparison of nonlinear interaction at intermodulation frequencies across conditions should be independent of any changes in responses at stimulus frequencies and their harmonics. In a control analysis, we implemented an established method for “correcting for” the potential influence of signals at stimulus frequencies and their harmonics, where we created tuning functions based on the ratio of power at I_1_ to the sum of power at stimulus frequencies 1F_A_ and 1F_B_ and their harmonics up to 40 Hz (see “Supplementary Information 9: Impact of power at stimulus frequencies on I_1_”). The shapes of tuning functions based on I_1_ did not change for any of our four experiments under this conservative analysis. We also note that typical patterns of amplitudes at I_1_ across color conditions are *opposite* to typical patterns of amplitudes at F_A_ and F_B_. Amplitudes at I_1_ are maximal at the smallest hue angle differences (e.g., 0°), while amplitudes at F_A_ and F_B_ are minimal. Thus, the changes in intermodulation we observe as a function of hue angle difference cannot be attributed to changes in SSVEP power at the stimulus frequencies and their harmonics.

Our investigation of intermodulation of EEG signals has revealed broadband cortical color tuning functions. We found that tuning is consistent across both cardinal and intermediate color axes, implying the action of intermediately tuned cortical color mechanisms. Surprisingly, the tuning functions we measured with intermodulation of EEG do not rely on chromatic edges, suggesting that they arise from neural populations that are not spatially selective. The bandwidths of our tuning functions are consistent with those derived psychophysically, from single-cell electrophysiology and from color masking in EEG. Intermodulation SSVEP is a promising method for probing cortical color representation in humans that reveals the action of different underlying chromatically responsive cell populations than those targeted by an existing method based on chromatic masking in conjunction with SSVEP.

## Supplementary Material

Supplementary Material

## Data Availability

All raw data and scripts necessary to process the data and reproduce the results presented in the paper are available via the Open Science Framework at https://osf.io/v5wxn/.
