# Supplementary material for: Tuning of cortical color mechanisms revealed using steady-state visually evoked potentials
Source: Imaging Neurosci (Camb). 2025 Aug 28;3:IMAG.a.130. doi: 10.1162/IMAG.a.130 (PMC12395283; doi:10.1162/IMAG.a.130)
Supplement: Supplementary Material [file IMAG.a.130_supp.pdf]

Supplementary Information

Supplementary Information 1: Tuning functions measured at different electrode scalp locations
One of the limitations of EEG as a neuroimaging method is its coarse spatial resolution. In an additional analysis, we investigated whether cortical color tuning functions change between scalp locations. We chose to separate the most posterior part of the scalp into three bands, each encompassing one set of electrodes, from the most posterior occipital cluster, through a parietal-occipital cluster to a parietal cluster, the most anterior of the three clusters (Supplementary Figure 1c).

For Experiments 1 and 4, data from all electrodes were extracted at I_1 (14.66 Hz) and $1F_B$ (8 Hz). The electrode signals were pre-processed as described in 'General methods' in the main paper. A single electrode exclusion (PO3) was made for one observer in Experiment 4.

Amplitudes were averaged across participants and electrodes in the sub-clusters (O, PO and P for occipital, parietal-occipital and parietal respectively). To compare the shapes of the tuning functions between clusters, the amplitudes were scaled to match in area under the curve. The close correspondence in the shapes of tuning functions based on amplitudes measured at electrodes in the 3 clusters is shown in Supplementary Figures 1a (Experiment 1) and 1b (Experiment 4). We interpret the matching shapes as evidence of transitivity in EEG signals where strong signals at one location can also be recorded in proximal channels. Consequently, we find no difference in tuning functions between posterior and more anterior sites. Imaging methods with more precise spatial resolution (such as fMRI) would be needed to determine differences in tuning between different cortical sites.

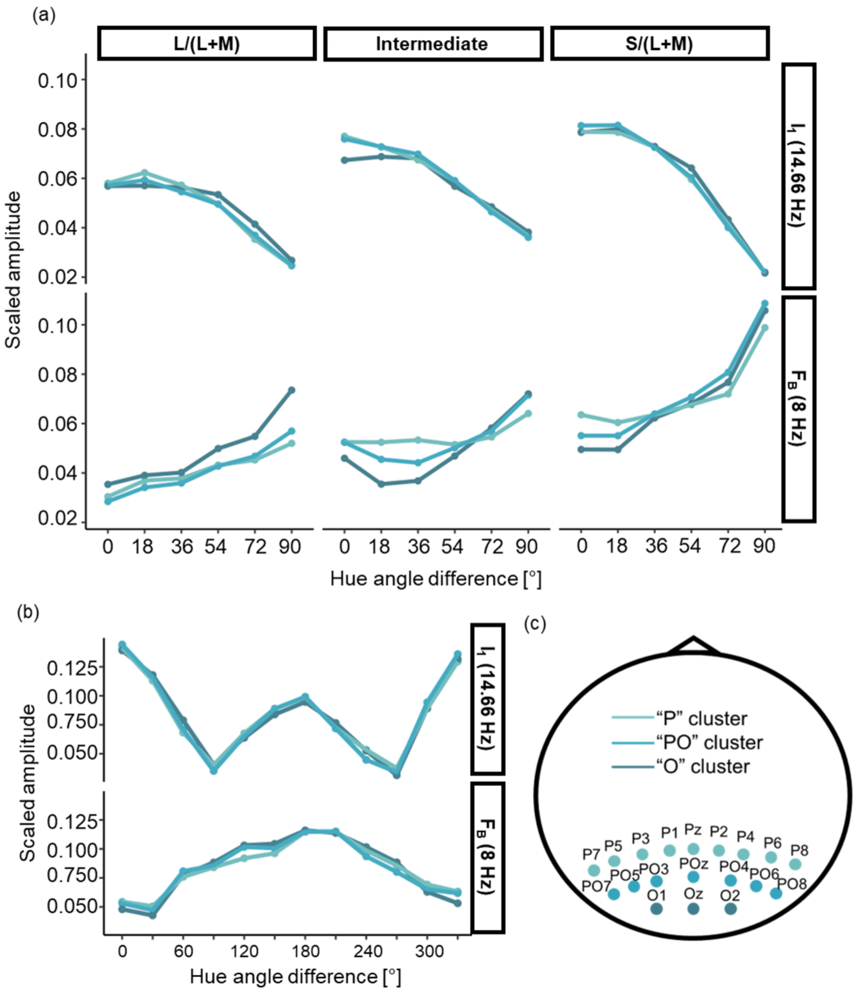

Supplementary Figure 1. Tuning functions based on amplitudes at different scalp locations. Panel (a) shows results from Experiment 1 and panel (b) shows results from Experiment 4. Each tuning function is scaled to match in area under the curve. (c) A schematic representation of the approximate scalp locations of electrodes in each cluster.

Supplementary Information 2: Treatment of eye-based artefacts

The results reported for Experiments 1-4 in the main paper are based on no inspection of trials for eye-based artefacts prior to further analysis. We conducted additional analyses for comparison, where eye-based artefacts were removed. If artefacts occurred on fewer than 20% of total trials in a condition, complete trials were removed. In cases where artefacts were present in more than 20% of the trials, independent component analysis (ICA) was performed and the identified components corresponding to eye-based artefacts were removed.

We present results of both approaches below. We investigated the effect of the two alternative approaches on final SNR for data for the S/(L+M) axis in Experiment 1. The data was analyzed and SNRs at I_1 (14.66 Hz) and $1F_B$ (8 Hz) were extracted for each of the two approaches.

Supplementary Figure 2 shows that the difference in SNR resulting from the two approaches to eye-based artefacts was minimal.

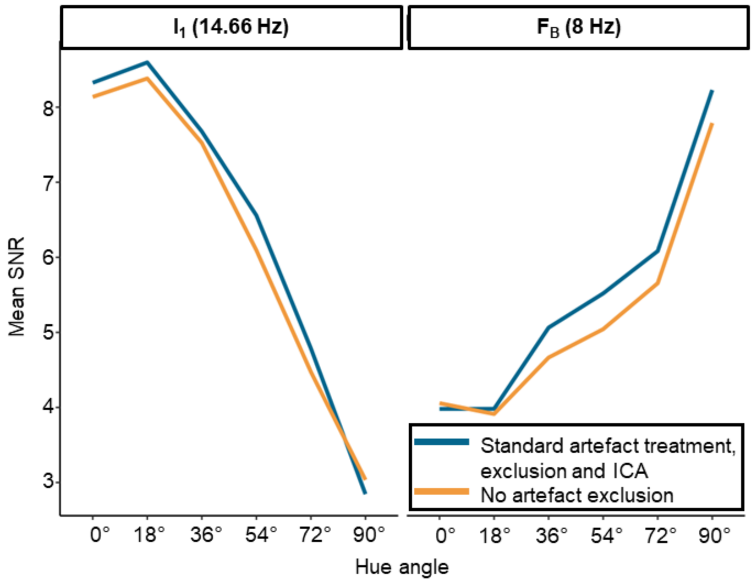

Supplementary Figure 2. Signal-to-noise ratios for the two ocular artefact treatments. The data in the example are from Experiment 1, for the S/(L+M) axis condition which was analyzed with both pipelines – either with ocular artefacts checked and excluded (blue line), or with no exclusions (orange line). The outcomes are presented as a function of hue angle difference between fixed and variable chromaticity, separately for I_1 (left) and $1F_B$ (right).

In Supplementary Figure 3 we plot the difference between the two approaches. This was derived by expressing the SNR for the pipeline without blink exclusions as percentage of the SNR for the pipeline with blink exclusions. If the two approaches completely matched in SNR, the percentage difference between them would be 0. Any positive difference shows higher SNR in for the pipeline where no artefacts were excluded, and a negative difference a higher SNR in the standard blink exclusion pipeline. For the S/(L+M) axis in Experiment 1, the absolute difference between blink-exclusion approaches did not surpass 10% in any condition. We thus concluded that the two approaches to eye-based artefact removal produce similar outcomes and used no eye-based artefact exclusion in our analyses.

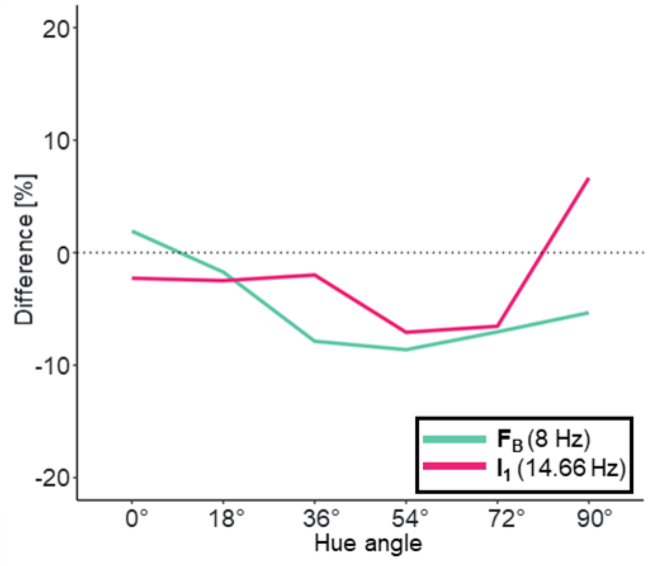

Supplementary Figure 3. Percentage difference in SNR between ocular artefact treatments in data processing. The difference in SNR between the no exclusions pipeline is expressed as percentage of the SNR in the standard exclusion pipeline. The data are from Experiment 1, from the S/(L+M) axis condition. The outcomes are presented as a function of hue angle difference between the fixed and variable chromaticities, separately for I_1 and $1F_B$. The dotted line denotes the point of no difference between pipelines (0% difference).

Supplementary Information 3: Post-hoc tests for Experiment 1

Supplementary Table 1. Post hoc paired-sample t-tests for the effect of hue angle difference on I_1 (14.66 Hz) in Experiment 1

		Mean Difference	SE	*t*	Cohen's *d*	p_{bonf}
0°	18°	0.007	0.050	0.132	0.020	1.000
	36°	0.050	0.050	1.000	0.150	1.000
	54°	0.135	0.050	2.725	0.409	0.133
	72°	0.273	0.050	5.510	0.828	< .001
	90°	0.403	0.050	8.139	1.223	< .001
18°	36°	0.043	0.050	0.868	0.130	1.000
	54°	0.128	0.050	2.593	0.390	0.187
	72°	0.266	0.050	5.378	0.808	< .001
	90°	0.396	0.050	8.007	1.203	< .001
36°	54°	0.085	0.050	1.725	0.259	1.000
	72°	0.223	0.050	4.510	0.678	< .001
	90°	0.353	0.050	7.138	1.073	< .001
54°	72°	0.138	0.050	2.785	0.419	0.113
	90°	0.268	0.050	5.414	0.814	< .001
72°	90°	0.130	0.050	2.629	0.395	0.170

Note. *p*-values have been Bonferroni-adjusted for 15 tests.

Supplementary Table 2. Post hoc paired-sample t-tests for the effect of axis on I_1 (14.66 Hz) in Experiment 1

		Mean Difference	SE	*t*	Cohen's *d*	p_{bonf}
L/(L+M)	Intermed.	-0.094	0.047	-2.013	-0.287	0.173
	S/(L+M)	-0.109	0.047	-2.318	-0.330	0.093
Intermed.	S/(L+M)	-0.014	0.047	-0.305	-0.043	1.000

Note. *p*-values have been Bonferroni-adjusted for 3 tests.

Supplementary Table 3. Post hoc paired-sample t-tests for the effect of hue angle difference on $1F_B$ in Experiment 1

		Mean Difference	SE	*t*	Cohen's *d*	p_{bonf}
0°	18°	0.016	0.029	0.573	0.068	1.000
	36°	-0.015	0.029	-0.513	-0.061	1.000
	54°	-0.057	0.029	-1.994	-0.237	0.774
	72°	-0.112	0.029	-3.888	-0.462	0.004
	90°	-0.244	0.029	-8.516	-1.012	< .001
18°	36°	-0.031	0.029	-1.086	-0.129	1.000
	54°	-0.074	0.029	-2.567	-0.305	0.199
	72°	-0.128	0.029	-4.461	-0.530	< .001
	90°	-0.261	0.029	-9.089	-1.080	< .001
36°	54°	-0.042	0.029	-1.481	-0.176	1.000
	72°	-0.097	0.029	-3.375	-0.401	0.022
	90°	-0.230	0.029	-8.003	-0.951	< .001
54°	72°	-0.054	0.029	-1.893	-0.225	0.961
	90°	-0.187	0.029	-6.522	-0.775	< .001
72°	90°	-0.133	0.029	-4.628	-0.550	< .001

Note. *p*-values have been Bonferroni-adjusted for 15 tests.

Supplementary Table 4. Post hoc paired-sample t-tests for the effect of axis on $1F_B$ in Experiment 1

		Mean Difference	SE	*t*	Cohen's *d*	p_{bonf}
L/(L+M)	Intermed.	-0.096	0.058	-1.655	-0.399	0.341
	S/(L+M)	-0.267	0.058	-4.595	-1.107	< .001
Intermed.	S/(L+M)	-0.171	0.058	-2.940	-0.708	0.024

Note. *p*-values have been Bonferroni-adjusted for 3 tests.

Supplementary Information 4: Post-hoc tests for Experiment 2

Supplementary Table 5. Post hoc paired-sample t-tests for the effect of hue angle difference on I_1 (14.66 Hz) in Experiment 2

		Mean Difference	SE	*t*	Cohen's *d*	p_{bonf}
0°	18°	0.017	0.018	0.926	0.053	1.000
	36°	0.059	0.025	2.397	0.189	0.562
	54°	0.139	0.044	3.200	0.444	0.142
	72°	0.246	0.070	3.518	0.783	0.083
	90°	0.350	0.095	3.680	1.115	0.064
18°	36°	0.043	0.019	2.267	0.136	0.702
	54°	0.123	0.040	3.080	0.390	0.175
	72°	0.229	0.070	3.268	0.730	0.127
	90°	0.334	0.097	3.436	1.062	0.096
36°	54°	0.080	0.028	2.828	0.254	0.269
	72°	0.187	0.064	2.930	0.594	0.226
	90°	0.291	0.095	3.079	0.926	0.175
54°	72°	0.107	0.041	2.573	0.340	0.416
	90°	0.211	0.077	2.725	0.672	0.321
72°	90°	0.104	0.040	2.580	0.332	0.411

Note. *p*-values have been Bonferroni-adjusted for 15 tests.

Supplementary Table 6. Post hoc paired-sample t-tests for the effect of hue angle difference on $1F_B$ (8 Hz) in Experiment 2

		Mean Difference	SE	*t*	Cohen's *d*	p_{bonf}
0°	18°	-0.061	0.044	-1.397	-0.126	1.000
	36°	-0.055	0.048	-1.164	-0.114	1.000
	54°	-0.036	0.045	-0.802	-0.075	1.000
	72°	-0.013	0.038	-0.334	-0.026	1.000
	90°	-0.029	0.044	-0.653	-0.059	1.000
18°	36°	0.006	0.042	0.135	0.012	1.000
	54°	0.025	0.052	0.474	0.051	1.000
	72°	0.048	0.053	0.913	0.099	1.000
	90°	0.032	0.051	0.631	0.067	1.000
36°	54°	0.019	0.025	0.760	0.039	1.000
	72°	0.042	0.041	1.041	0.087	1.000
	90°	0.027	0.040	0.676	0.055	1.000
54°	72°	0.023	0.028	0.844	0.048	1.000
	90°	0.008	0.022	0.353	0.016	1.000
72°	90°	-0.016	0.018	-0.888	-0.032	1.000

Note. *p*-values have been Bonferroni-adjusted for 15 tests.

Supplementary Information 5: Post-hoc tests for Experiment 3

Supplementary Table 7. Post hoc paired-sample t-tests for the effect of check size on amplitudes at I_1 (14.66 Hz) in Experiment 3

		Mean Difference	SE	*t*	Cohen's *d*	p_{bonf}
.096°	.4°	-0.002	0.040	-0.041	-0.005	1.000
	1.3°	-0.156	0.034	-4.575	-0.502	0.006
	4.8°	8.974×10^{-4}	0.044	0.020	0.003	1.000
.4°	1.3°	-0.154	0.037	-4.164	-0.497	0.012
	4.8°	0.003	0.034	0.075	0.008	1.000
1.3°	4.8°	0.157	0.046	3.398	0.505	0.041

Note. *p*-values have been Bonferroni-adjusted for 6 tests.

Supplementary Table 8. Post hoc paired-sample t-tests for the effect of hue angle difference on amplitudes at I_1 (14.66 Hz) in Experiment 3

		Mean Difference	SE	*t*	Cohen's *d*	p_{bonf}
0°	30°	0.095	0.016	5.761	0.305	0.001
	60°	0.308	0.057	5.445	0.991	0.002
	90°	0.494	0.090	5.474	1.591	0.002
30°	60°	0.213	0.044	4.836	0.686	0.004
	90°	0.399	0.081	4.912	1.286	0.004
60°	90°	0.186	0.042	4.449	0.600	0.007

Note. *p*-values have been Bonferroni-adjusted for 6 tests.

Supplementary Table 9. Post hoc paired-sample t-tests for the effect of check size on amplitudes at $1F_B$ (8 Hz) in Experiment 3

		Mean Difference	SE	*t*	Cohen's *d*	p_{bonf}
.096°	.4°	0.034	0.038	0.896	0.099	1.000
	1.3°	0.038	0.054	0.693	0.110	1.000
	4.8°	0.059	0.047	1.248	0.172	1.000
.4°	1.3°	0.004	0.077	0.049	0.011	1.000
	4.8°	0.025	0.073	0.339	0.073	1.000
1.3°	4.8°	0.021	0.028	0.756	0.062	1.000

Note. *p*-values have been Bonferroni-adjusted for 6 tests.

Supplementary Table 10. Post hoc paired-sample t-tests for the effect of hue angle difference on amplitudes at $1F_B$ (8 Hz) in Experiment 3

		Mean Difference	SE	*t*	Cohen's *d*	p_{bonf}
0°	30°	-0.017	0.026	-0.644	-0.049	1.000
	60°	-0.073	0.031	-2.364	-0.214	0.238
	90°	-0.310	0.071	-4.357	-0.906	0.009
30°	60°	-0.056	0.033	-1.691	-0.165	0.730
	90°	-0.293	0.076	-3.858	-0.858	0.019
60°	90°	-0.236	0.056	-4.247	-0.692	0.010

Note. *p*-values have been Bonferroni-adjusted for 6 tests.

Supplementary Information 6: Post-hoc tests for Experiment 4

Supplementary Table 11. Post hoc paired-sample t-tests for the effect of hue angle difference on amplitudes at I_1 (14.66 Hz) in Experiment 4

		Mean Difference	SE	*t*	Cohen's *d*	p_{bonf}
0°	30°	0.255	0.114	2.223	0.664	1.000
	60°	0.671	0.114	5.866	1.751	< .001
	90°	1.021	0.114	8.920	2.662	< .001
	120°	0.756	0.114	6.604	1.971	< .001
	150°	0.552	0.114	4.822	1.439	< .001
	180°	0.450	0.114	3.935	1.174	0.010
	210°	0.677	0.114	5.915	1.765	< .001
	240°	0.928	0.114	8.109	2.420	< .001
	270°	1.055	0.114	9.215	2.750	< .001
	300°	0.489	0.114	4.268	1.274	0.003
	330°	0.087	0.114	0.756	0.226	1.000
30°	60°	0.417	0.114	3.642	1.087	0.029
	90°	0.767	0.114	6.697	1.999	< .001
	120°	0.502	0.114	4.381	1.308	0.002
	150°	0.297	0.114	2.598	0.776	0.712
	180°	0.196	0.114	1.711	0.511	1.000
	210°	0.423	0.114	3.692	1.102	0.024
	240°	0.674	0.114	5.886	1.757	< .001
	270°	0.800	0.114	6.992	2.087	< .001
	300°	0.234	0.114	2.045	0.610	1.000
	330°	-0.168	0.114	-1.467	-0.438	1.000
60°	90°	0.350	0.114	3.054	0.912	0.191
	120°	0.085	0.114	0.739	0.221	1.000
	150°	-0.120	0.114	-1.044	-0.312	1.000
	180°	-0.221	0.114	-1.931	-0.576	1.000
	210°	0.006	0.114	0.050	0.015	1.000
	240°	0.257	0.114	2.244	0.670	1.000
	270°	0.383	0.114	3.349	1.000	0.076
	300°	-0.183	0.114	-1.598	-0.477	1.000
	330°	-0.585	0.114	-5.110	-1.525	< .001
90°	120°	-0.265	0.114	-2.316	-0.691	1.000
	150°	-0.469	0.114	-4.099	-1.223	0.006
	180°	-0.571	0.114	-4.985	-1.488	< .001
	210°	-0.344	0.114	-3.005	-0.897	0.222
	240°	-0.093	0.114	-0.811	-0.242	1.000
	270°	0.034	0.114	0.295	0.088	1.000
	300°	-0.533	0.114	-4.652	-1.389	< .001
	330°	-0.935	0.114	-8.164	-2.437	< .001
120°	150°	-0.204	0.114	-1.783	-0.532	1.000
	180°	-0.306	0.114	-2.670	-0.797	0.585
	210°	-0.079	0.114	-0.689	-0.206	1.000
	240°	0.172	0.114	1.505	0.449	1.000

	270°	0.299	0.114	2.611	0.779	0.689
	300°	-0.268	0.114	-2.337	-0.697	1.000
	330°	-0.670	0.114	-5.849	-1.746	< .001
150°	180°	-0.102	0.114	-0.887	-0.265	1.000
	210°	0.125	0.114	1.094	0.326	1.000
	240°	0.376	0.114	3.288	0.981	0.092
	270°	0.503	0.114	4.394	1.311	0.002
	300°	-0.063	0.114	-0.554	-0.165	1.000
	330°	-0.465	0.114	-4.066	-1.213	0.006
180°	210°	0.227	0.114	1.981	0.591	1.000
	240°	0.478	0.114	4.175	1.246	0.004
	270°	0.605	0.114	5.280	1.576	< .001
	300°	0.038	0.114	0.333	0.099	1.000
	330°	-0.364	0.114	-3.179	-0.949	0.130
210°	240°	0.251	0.114	2.194	0.655	1.000
	270°	0.378	0.114	3.300	0.985	0.089
	300°	-0.189	0.114	-1.647	-0.492	1.000
	330°	-0.591	0.114	-5.159	-1.540	< .001
240°	270°	0.127	0.114	1.106	0.330	1.000
	300°	-0.440	0.114	-3.841	-1.147	0.014
	330°	-0.842	0.114	-7.353	-2.195	< .001
270°	300°	-0.566	0.114	-4.947	-1.477	< .001
	330°	-0.968	0.114	-8.459	-2.525	< .001
300°	330°	-0.402	0.114	-3.512	-1.048	0.044

Note. *p*-values have been Bonferroni-adjusted for 66 tests.

Supplementary Table 12. Post hoc paired-sample t-tests for the effect of hue angle difference on amplitudes $1F_B$ (8 Hz) in Experiment 4

		Mean Difference	SE	*t*	Cohen's *d*	p_{bonf}
0°	30°	0.074	0.125	0.590	0.168	1.000
	60°	-0.303	0.125	-2.416	-0.689	1.000
	90°	-0.419	0.125	-3.338	-0.953	0.078
	120°	-0.570	0.125	-4.544	-1.297	0.001
	150°	-0.587	0.125	-4.685	-1.337	< .001
	180°	-0.733	0.125	-5.849	-1.669	< .001
	210°	-0.721	0.125	-5.751	-1.641	< .001
	240°	-0.541	0.125	-4.311	-1.230	0.003
	270°	-0.392	0.125	-3.125	-0.892	0.154
	300°	-0.132	0.125	-1.055	-0.301	1.000
	330°	-0.083	0.125	-0.661	-0.189	1.000
30°	60°	-0.377	0.125	-3.006	-0.858	0.222
	90°	-0.493	0.125	-3.928	-1.121	0.010
	120°	-0.644	0.125	-5.134	-1.465	< .001
	150°	-0.661	0.125	-5.275	-1.505	< .001

	180°	-0.807	0.125	-6.439	-1.838	< .001
	210°	-0.795	0.125	-6.341	-1.810	< .001
	240°	-0.615	0.125	-4.901	-1.399	< .001
	270°	-0.466	0.125	-3.715	-1.060	0.022
	300°	-0.206	0.125	-1.645	-0.469	1.000
	330°	-0.157	0.125	-1.251	-0.357	1.000
60°	90°	-0.116	0.125	-0.923	-0.263	1.000
	120°	-0.267	0.125	-2.128	-0.607	1.000
	150°	-0.285	0.125	-2.269	-0.648	1.000
	180°	-0.430	0.125	-3.433	-0.980	0.058
	210°	-0.418	0.125	-3.336	-0.952	0.079
	240°	-0.238	0.125	-1.896	-0.541	1.000
	270°	-0.089	0.125	-0.709	-0.202	1.000
	300°	0.171	0.125	1.361	0.388	1.000
	330°	0.220	0.125	1.755	0.501	1.000
90°	120°	-0.151	0.125	-1.205	-0.344	1.000
	150°	-0.169	0.125	-1.347	-0.384	1.000
	180°	-0.315	0.125	-2.511	-0.717	0.902
	210°	-0.303	0.125	-2.413	-0.689	1.000
	240°	-0.122	0.125	-0.973	-0.278	1.000
	270°	0.027	0.125	0.213	0.061	1.000
	300°	0.286	0.125	2.283	0.652	1.000
	330°	0.336	0.125	2.677	0.764	0.573
120°	150°	-0.018	0.125	-0.141	-0.040	1.000
	180°	-0.164	0.125	-1.305	-0.373	1.000
	210°	-0.151	0.125	-1.208	-0.345	1.000
	240°	0.029	0.125	0.232	0.066	1.000
	270°	0.178	0.125	1.419	0.405	1.000
	300°	0.437	0.125	3.489	0.996	0.048
	330°	0.487	0.125	3.883	1.108	0.012
150°	180°	-0.146	0.125	-1.164	-0.332	1.000
	210°	-0.134	0.125	-1.066	-0.304	1.000
	240°	0.047	0.125	0.374	0.107	1.000
	270°	0.196	0.125	1.560	0.445	1.000
	300°	0.455	0.125	3.630	1.036	0.030
	330°	0.505	0.125	4.024	1.148	0.007
180°	210°	0.012	0.125	0.098	0.028	1.000
	240°	0.193	0.125	1.538	0.439	1.000
	270°	0.342	0.125	2.724	0.777	0.503
	300°	0.601	0.125	4.794	1.368	< .001
	330°	0.650	0.125	5.188	1.481	< .001
210°	240°	0.181	0.125	1.440	0.411	1.000
	270°	0.329	0.125	2.626	0.750	0.660
	300°	0.589	0.125	4.696	1.340	< .001
	330°	0.638	0.125	5.090	1.453	< .001
240°	270°	0.149	0.125	1.186	0.339	1.000
	300°	0.408	0.125	3.256	0.929	0.102

	330°	0.458	0.125	3.650	1.042	0.028
270°	300°	0.260	0.125	2.070	0.591	1.000
	330°	0.309	0.125	2.464	0.703	1.000
300°	330°	0.049	0.125	0.394	0.112	1.000

Note. *p*-values have been Bonferroni-adjusted for 66 tests.

Supplementary information 7: Scalp maps for Experiments 1 and 4

We extracted signals at all scalp locations for Experiments 1 and 4. We present these as scalp heatmap plots in Supplementary Figures 4 and 5, respectively.

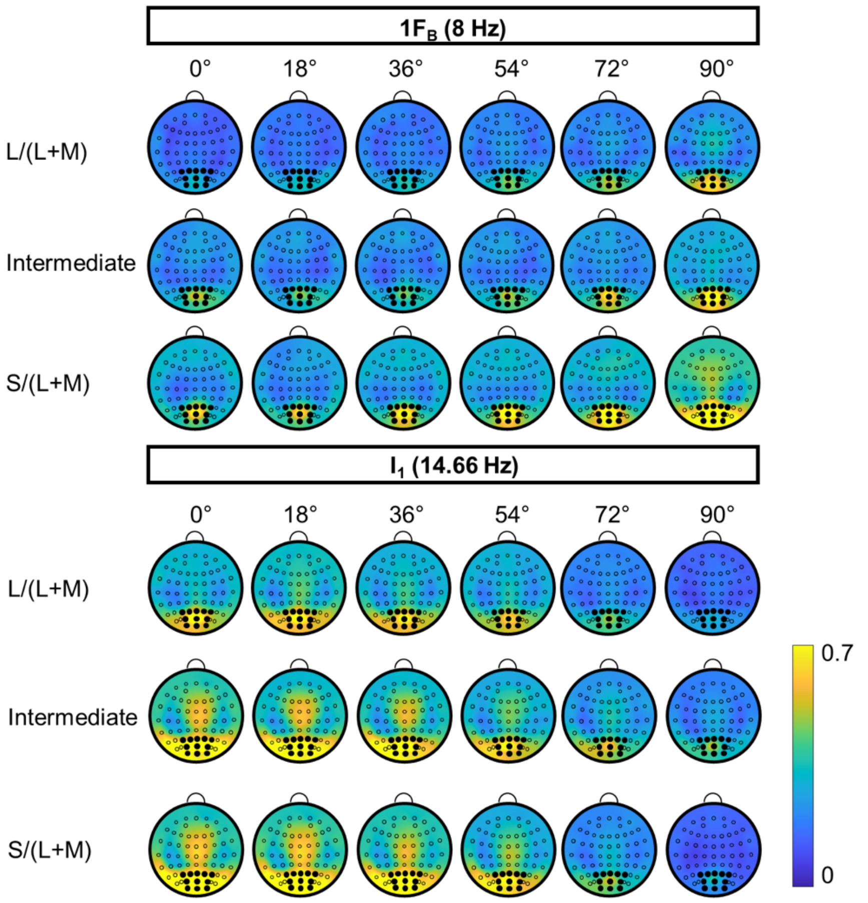

Supplementary Figure 4. Heatmaps of average amplitudes in Experiment 1. The amplitudes reflect the average across participants. Electrode positions are marked by open circles and the electrode cluster used in our results section is marked by the closed circles.

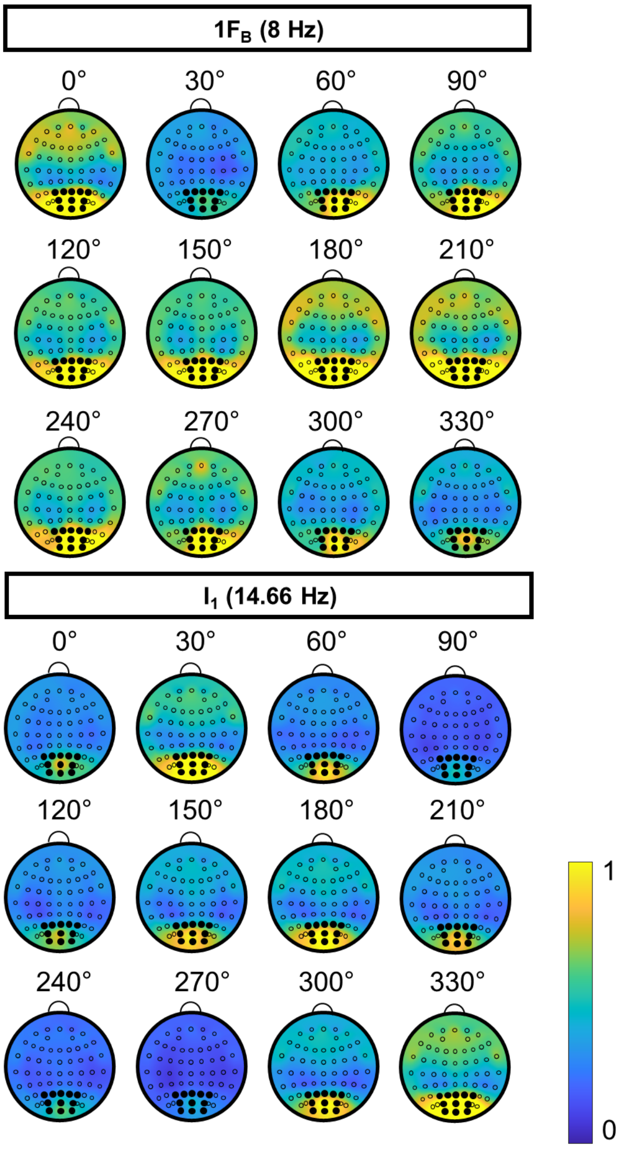

Supplementary Figure 5. Heatmaps of average amplitudes in Experiment 4. The amplitudes reflect the average across participants. Electrode positions are marked by open circles and the electrode cluster used in our results section is marked by the closed circles.

Supplementary Information 8: Split-half reliability of tuning functions from Experiment 4.

We performed a split-half reliability analysis of the results of Experiment 4. Here, we randomly selected half of the trials for each participant and used them to plot a tuning function as described in Section 6.2 of the main paper. We plotted a second tuning function based on the other random half of trials. We plot the two tuning functions based on split-halves of the data in Supplementary Figure 6. The figure shows that tuning functions derived from I_1 and from $1F_B$ are highly reliable, as their shapes are very consistent when based on split halves of the data. We calculated ICC(C,1) correlation coefficients, entering into the correlation a vector of all participant/condition mean amplitudes per split half of trials. The ICC(C,1) for tuning functions based on I_1 was 0.9031 (95% confidence interval: 0.79-0.96). The ICC(C,2) for tuning functions based on 1FB was 0.9022 (95% confidence interval: 0.86-0.93). Rozman et al. (2025) provide a more in-depth analysis of reliability.

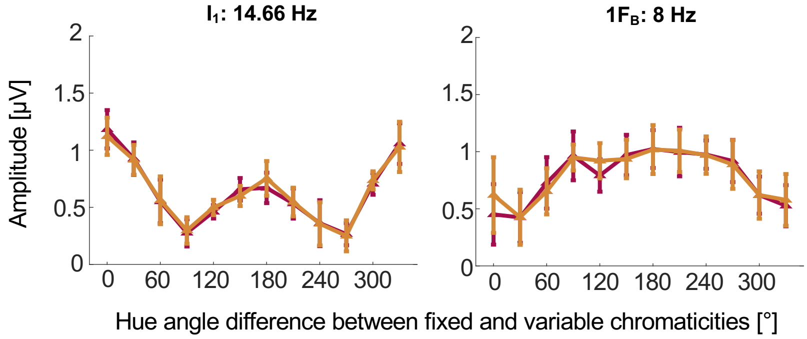

Supplementary Figure 6. Tuning functions based on split halves of the data from Experiment 4. Plotted in red are tuning functions based on one randomly selected half of trials for each participant. Plotted in orange are tuning functions based on the other half of trials. Error bars indicate within-participant 95% confidence intervals.

Supplementary Information 9: Impact of power at stimulus frequencies on I_1

Signals at the intermodulation frequency I_1 could be impacted by power at fundamental stimulus frequencies F_A and F_B and/or their harmonics (Gordon et al., 2019; Zhang et al., 2011). We performed an analysis to plot tuning functions based on I_1 'corrected' for power at the stimulus frequencies and their harmonics (Rozman et al., 2025). For each of the four experiments, we normalized power at the I_1 intermodulation frequency by the sum of powers at F_A (6.66 Hz) and F_B (8 Hz) and their harmonics ≤40 Hz. For each target fundamental or harmonic frequency (6.66, 13.33, 19.99, 26.66 and 33.33 for F_A; 8,16,24,32 and 40 for F_B) an amplitude was extracted, together with amplitudes in 5 neighbouring frequency bins to each side (5 before and 5 after the target frequency). The minimum and maximum values were excluded from this 10-bin baseline, and baseline amplitude was calculated as a mean of the remaining 8 bins for each target frequency. The baseline amplitude was then subtracted from all frequencies, including the target frequency, producing a baseline-corrected peak amplitude at the target frequency. We then converted from amplitude to power by squaring (μV^2). Baseline-corrected powers were then summed for F_A and F_B and their harmonics. The same procedure was applied to I_1. The power value at baseline-corrected I_1 was then divided by the summed power at baseline-corrected F_A and F_B and their harmonics to obtain a power ratio for each hue angle difference condition. Tuning functions for experiments 1-4 based on these power ratios are presented in Supplementary Figures 7-10, together with tuning functions based on mean amplitudes as presented in the main paper. While the power normalization alters the precise forms of the tuning functions, their salient properties are preserved, i.e., the reduction in amplitude or power ratio with increasing hue angle difference in tuning functions measured in Experiments 1-3, and the secondary peak in the tuning function measured in Experiment 4. We conclude that the shapes of tuning functions based on amplitudes at I_1 presented in the main paper cannot be explained by power at stimulus frequencies or their harmonics.

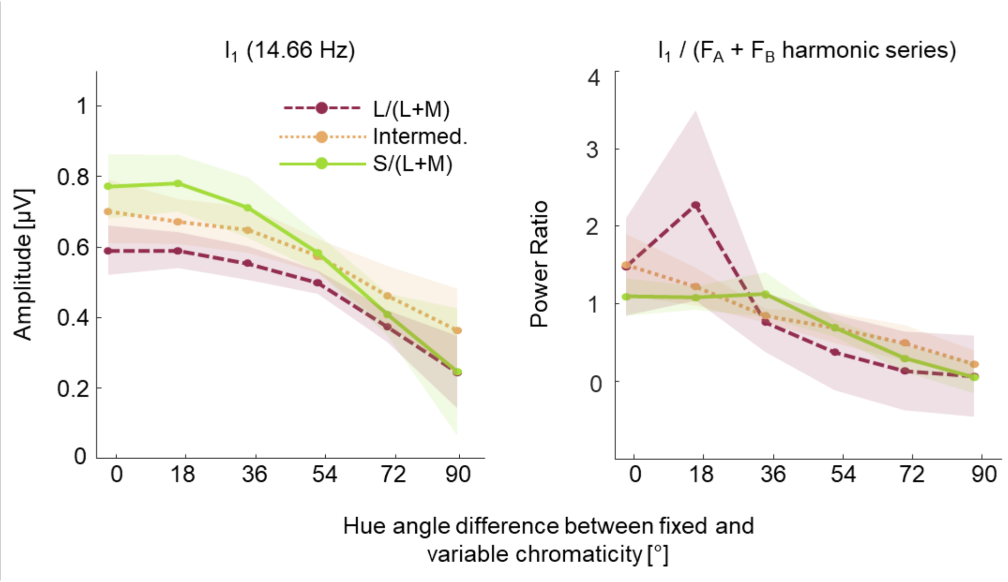

Supplementary Figure 7. Tuning functions for Experiment 1. In the left panel (replotted from Figure 2), amplitudes at I_1 (14.66 Hz) are plotted as a function of hue angle difference. In the right panel a ratio of power at I_1 to power at the sum of the harmonic series for the stimulus frequencies (F_A and F_B) is plotted as a function of hue angle difference. In both panels, lines show mean values across participants and error clouds show 95% within-participant confidence intervals. The dashed red, dotted orange and solid green lines show data for stimuli centered on the L/(L+M) axis, the intermediate axis, and the S/(L+M) axis, respectively.

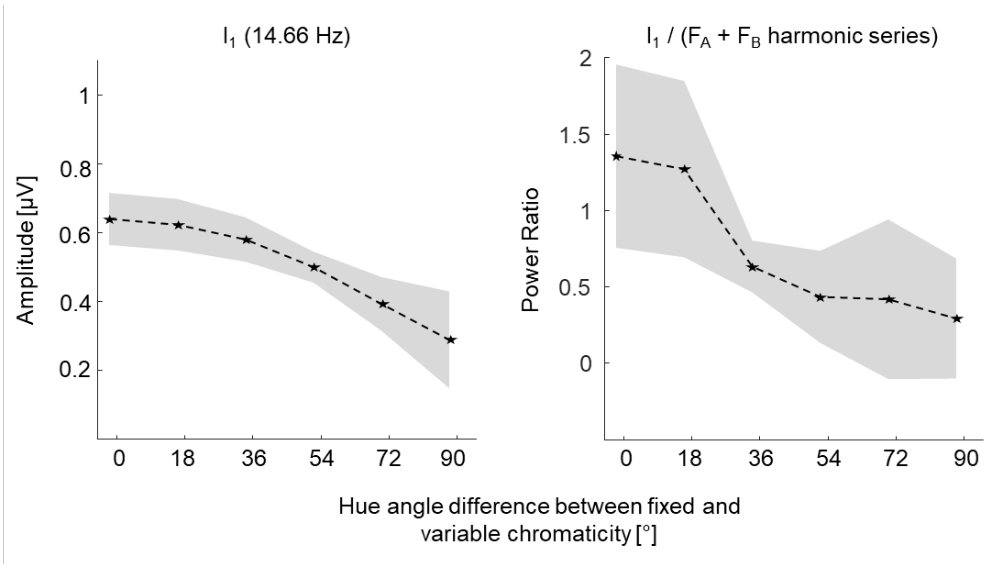

Supplementary Figure 8. Tuning functions for Experiment 2. In the left panel (replotted from Figure 3), amplitudes at I_1 (14.66 Hz) are plotted as a function of hue angle difference. In the right panel a ratio of power at I_1 to power at the sum of the harmonic series for the stimulus frequencies (F_A and F_B) is plotted as a function of hue angle difference. In both panels, lines show mean values across participants and error clouds show 95% within-participant confidence intervals.

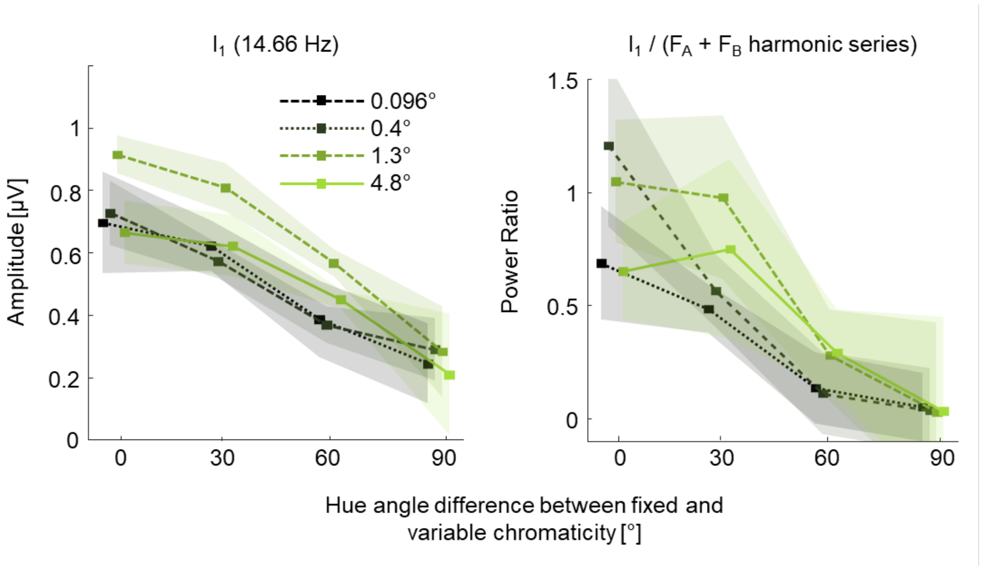

Supplementary Figure 9. Tuning functions for Experiment 3. In the left panel (replotted from Figure 4), amplitudes at I_1 (14.66 Hz) are plotted as a function of hue angle difference. In the right panel a ratio of power at I_1 to power at the sum of the harmonic series for the stimulus frequencies (F_A and F_B) is plotted as a function of hue angle difference. In both panels, lines show mean values across participants and error clouds show 95% within-participant confidence intervals. Dashed black lines show results for the 0.096° check size condition, dotted black lines for the 0.4° check size condition, dashed green lines for the 1.3° check size condition, and full green lines for the 4.8° check size condition.

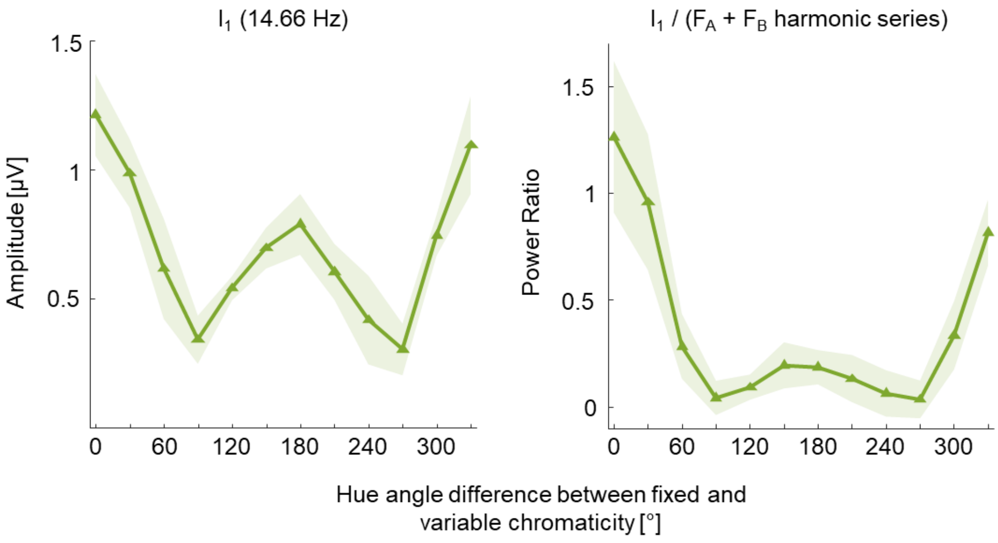

Supplementary Figure 10. Tuning functions for Experiment 4. In the left panel (replotted from Figure 5), amplitudes at I_1 (14.66 Hz) are plotted as a function of hue angle difference. In the right panel a ratio of power at I_1 to power at the sum of the harmonic series for the stimulus frequencies (F_A and F_B) is plotted as a function of hue angle difference. In both panels, lines show mean values across participants and error clouds show 95% within-participant confidence intervals.

References

Gordon, N., Hohwy, J., Davidson, M. J., Van Boxtel, J. J. A., & Tsuchiya, N. (2019). From intermodulation components to visual perception and cognition-a review. *NeuroImage*, *199*, 480–494. https://doi.org/10.1016/j.neuroimage.2019.06.008

Rozman, A., Racey, C., & Bosten, J. M. (2025). Comparison of intermodulation and oddball methods for measuring human cortical color tuning functions using steady-state visually evoked potentials. *JOSA A*, *42*(5), B335–B345. https://doi.org/10.1364/JOSAA.545280

Zhang, P., Jamison, K., Engel, S., He, B., & He, S. (2011). Binocular Rivalry Requires Visual Attention. *Neuron*, *71*(2), 362–369. https://doi.org/10.1016/j.neuron.2011.05.035
